# Michael Acceptors as Anti-Cancer Compounds: Coincidence or Causality?

**DOI:** 10.3390/ijms25116099

**Published:** 2024-06-01

**Authors:** Celia María Curieses Andrés, José Manuel Pérez de la Lastra, Elena Bustamante Munguira, Celia Andrés Juan, Eduardo Pérez-Lebeña

**Affiliations:** 1 Hospital Clínico Universitario of Valladolid, Avenida de Ramón y Cajal, 3, 47003 Valladolid, Spain; cmcurieses@gmail.com (C.M.C.A.); ebustamante@saludcastillayleon.es (E.B.M.); 2 Institute of Natural Products and Agrobiology, CSIC-Spanish Research Council, Avda. Astrofísico Fco. Sánchez, 3, 38206 La Laguna, Spain; 3 Cinquima Institute and Department of Organic Chemistry, Faculty of Sciences, Valladolid University, Paseo de Belén, 7, 47011 Valladolid, Spain; celia.andres.juan@uva.es; 4 Sistemas de Biotecnología y Recursos Naturales, 47625 Valladolid, Spain; info@glize.eu

**Keywords:** Michael acceptor compounds, neoplastic metabolism, chemotherapy

## Abstract

Michael acceptors represent a class of compounds with potential anti-cancer properties. They act by binding to nucleophilic sites in biological molecules, thereby disrupting cancer cell function and inducing cell death. This mode of action, as well as their ability to be modified and targeted, makes them a promising avenue for advancing cancer therapy. We are investigating the molecular mechanisms underlying Michael acceptors and their interactions with cancer cells, in particular their ability to interfere with cellular processes and induce apoptosis. The anti-cancer properties of Michael acceptors are not accidental but are due to their chemical structure and reactivity. The electrophilic nature of these compounds allows them to selectively target nucleophilic residues on disease-associated proteins, resulting in significant therapeutic benefits and minimal toxicity in various diseases. This opens up new perspectives for the development of more effective and precise cancer drugs. Nevertheless, further studies are essential to fully understand the impact of our discoveries and translate them into clinical practice.

## 1. Introduction

In organic chemistry, a Michael acceptor (MA) usually refers to an α,β-unsaturated carbonyl compound commonly used in the Michael reaction or Michael 1,4 addition. This reaction involves the interaction between a Michael donor, which acts as a nucleophile and an MA. Various unsaturated carbonyl compounds such as aldehydes, esters, thioesters, nitriles, amides, and nitro compounds can act as electrophilic acceptor components in Michael reactions.

Post-translational modifications (PTMs) are a fundamental mechanism for regulating protein function by altering their physical and chemical properties, folding, conformation, stability and activity. The best-known PTMs include phosphorylation, acetylation, ubiquitination, methylation, oxidation and glycosylation [1], with different functions:

- Phosphorylation regulates numerous cellular processes such as the cell cycle, and growth and signal transduction pathways [2].
- Acetylation primarily affects protein stability and interactions and often occurs at the N-terminus [3].
- Ubiquitination signals protein degradation [4].
- Methylation alters the interactions of proteins with DNA, RNA or other proteins [5].
- Glycosylation affects the folding, stability and cell adhesion of proteins [6].
- Oxidation occurs when reactive oxygen species (ROS) donate electrons to proteins. It can extensively modify the primary structure of proteins and peptides and often leads to modifications of higher-order structures. It is implicated in human disease, carcinogenesis and aging [7].

These modifications can occur at any point in the life cycle of a protein and can be reversible or irreversible. The specific pattern of PTMs, often referred to as the ‘PTM code’, significantly influences the biological function of a protein and emphasizes the importance of understanding PTMs for the elucidation of biological processes and disease mechanisms at the molecular level [8].

Cysteine residues in proteins play a crucial role in covalent modifications due to their unique reactivity, especially the high reactivity of the thiol group. Cysteine can undergo various PTMs, including palmitoylation, glutathionylation, guanylation, cysteinylation, nitrosylation and sulfhydrogenation, which affect the structure, reactivity, stability and function of proteins and thereby modulate various biological processes [9].

In addition, cysteine side chains can form disulfide bonds, an important biochemical mechanism that nature uses to influence the function of macromolecules. Furthermore, both endogenous and exogenous electrophilic molecules can covalently modify nucleophilic cysteine residues and thus alter the state and functions of cells [10].

MA molecules that have biologically active electrophilic groups in their structure can selectively target nucleophilic residues of disease-relevant proteins, leading to significant therapeutic effects with low toxicity in various diseases. These molecules, often derived from plants, exert their effects via signaling pathways such as Keap1-NRF2-ARE and NF-κB [11]. They show biological activities such as antioxidant and anti-inflammatory effects and are therefore promising for the treatment of diseases associated with oxidative stress [12], inflammation and cancer [13].

In addition, nitro fatty acids (NFAs), another type of MA, have shown therapeutic efficacy in animal models of inflammation and cancer, leading to Phase II clinical trials investigating their potential therapeutic effect in patients with pulmonary arterial hypertension [14].

In this manuscript, we have carried out a systematic review of the mechanism of action of covalent binding to proteins and MA molecules and their relationship with cancer treatments, both in synthetic and natural substances. Many MA compounds derived from plants have significant therapeutic effects and low toxicity for many diseases, including cancer [13]. With this work, we have tried to contribute to the understanding of the scope of this mechanism in the treatment of various diseases, especially cancer.

## 2. Michael Reaction and Covalent Binding to Proteins

### 2.1. Michael Reaction

**Figure 1:**
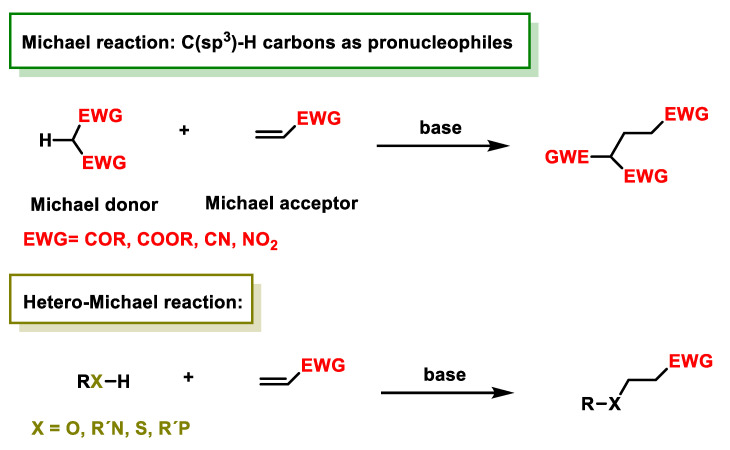
Michael reaction utilizing different (pro-)nucleophiles.

The Michael reaction, in which an enolate is added to an activated alkene typically represented by an α,β-unsaturated carbonyl compound, is considered one of the most efficient methods for forming C-C bonds [15,16]. Originally discovered by Arthur Michael in the late 1880s, this reaction has been extensively researched and led to the development of numerous reaction variants [17,18,19,20,21,22,23]. Among these, hetero-Michael additions such as oxa- [24,25,26,27], aza- [28,29], thia- [30,31,32,33,34] and phospha-Michael reactions [35] represent extensions of this concept to heterocentric anions (Figure 1).

**Figure 2:**
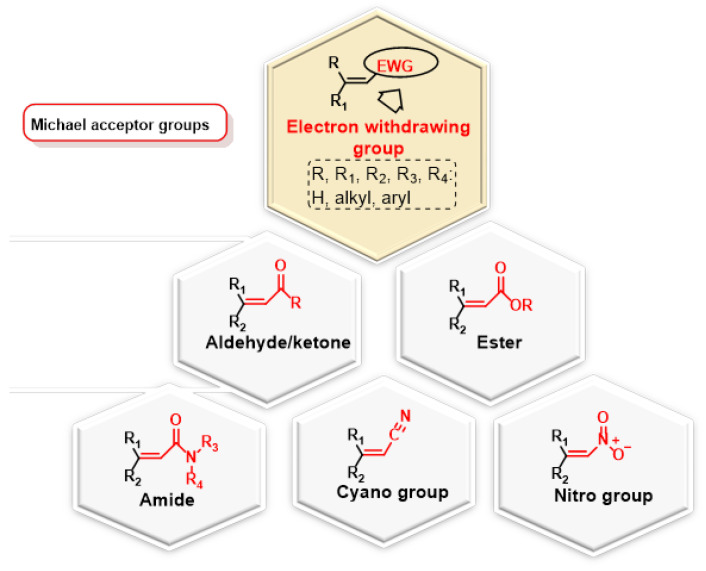
Michael acceptor groups.

The nucleophiles used in the Michael reaction include substrates with C(sp3)-H bonds coupled with electron-accepting groups such as acyl and cyano, which increase the acidity of the methylene hydrogens and thus facilitate the formation of carbanions by deprotonation with a suitable base. Substrates with H-hetero groups such as ROH, R2NH, RSH and R2PHAs are also used (Figure 2).

**Figure 3:**
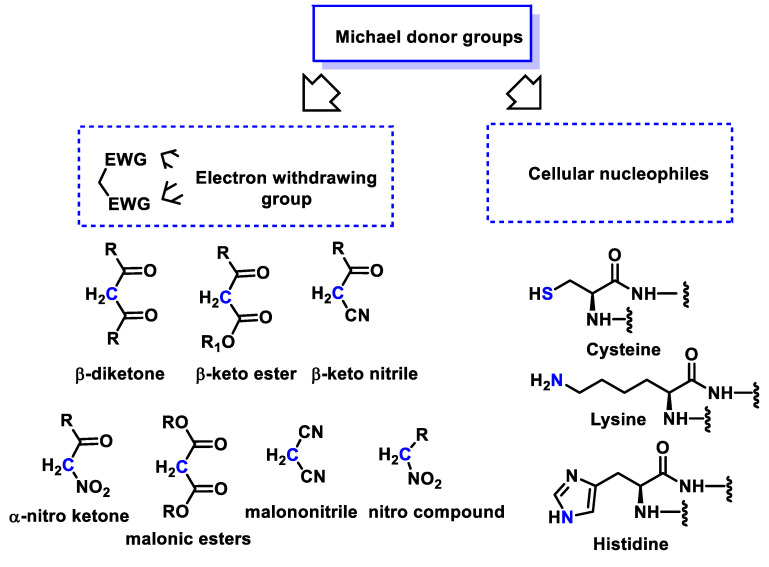
Michael donor groups.

Common examples of nucleophiles are beta-ketoesters, malonates and beta-cyanoesters. In addition, the carbonyl group within the α,β-unsaturated carbonyl compound can be replaced by a nitro group or another strong electron acceptor group. Important nucleophilic groups such as the thiol group of cysteine, the imidazole group of histidine or the ε-amino group of lysine serve as Michael donors (Figure 3).

MA molecules can form complexes with nucleophilic protein residues and thereby modulate protein pathways to exert physiological effects. They play a central role in the regulation of signaling pathways such as Keap1-NRF2-ARE and NF-κB, making them promising candidates for the treatment of various diseases such as inflammation, cancer and oxidative stress [36].

**Figure 4:**
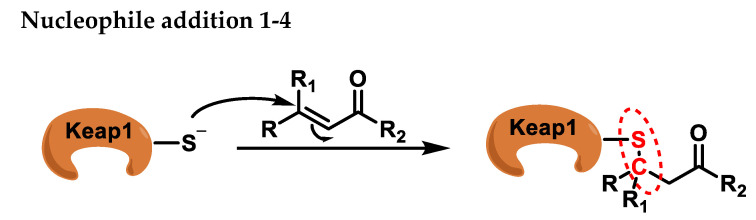
MA reaction with the thiol group of cysteines in Keap1 protein.

MA compounds can undergo addition reactions with the thiol group of cysteines, whereby the sulfur of the cysteine residue is added to an activated carbon of the MA compound [37], typically through a nucleophilic 1-4 addition reaction. This reaction, which falls into the realm of conjugate additions, provides a mild method for the S-C bond formation (Figure 4).

By altering the biological conformation of proteins or enzymes containing nucleophilic groups, MA compounds induce physiological changes, some of which are approved as drugs for various therapeutic treatments [38]. Many naturally occurring compounds such as piperine, acetogenins, leptomycin B, certain β-carotenes (e.g., fucoxanthin and astaxanthin), curcuminoids, chalcones, coumarins and terpenoids contain MA groups in their structures or derivatives that make them biologically active. These compounds react with nucleophilic residues in proteins and provide significant therapeutic effects with minimal toxicity for various diseases [13].

### 2.2. The Kinetics of Inhibition via Michael Reaction

**Figure 5:**
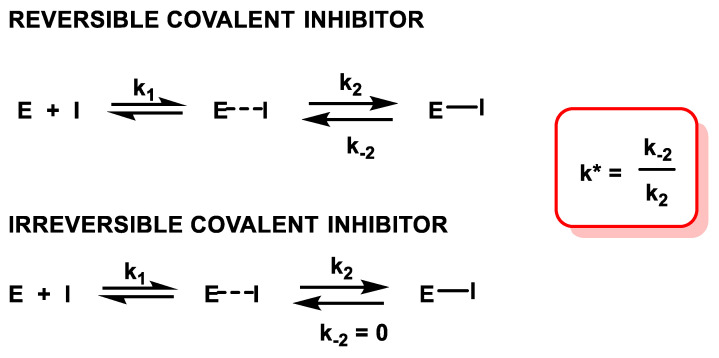
Mechanism of reversible and irreversible covalent inhibitors.

Covalent binding to proteins can be reversible or irreversible and there are important differences between irreversible covalent inhibitors and reversible covalent inhibitors. The rate of covalent bond formation (E-I) is relatively slow, and it is necessary to consider the value of the reaction equilibrium constant k* to know if it is an irreversible covalent inhibitor (k_-2_ = 0 or close to zero), in this case k* tends to infinity. When the difference between k_2_ and k_-2_ is not very large, i.e., when k* is in a reasonable range, it can be called a reversible covalent bond [39] (Figure 5).

**Figure 6:**
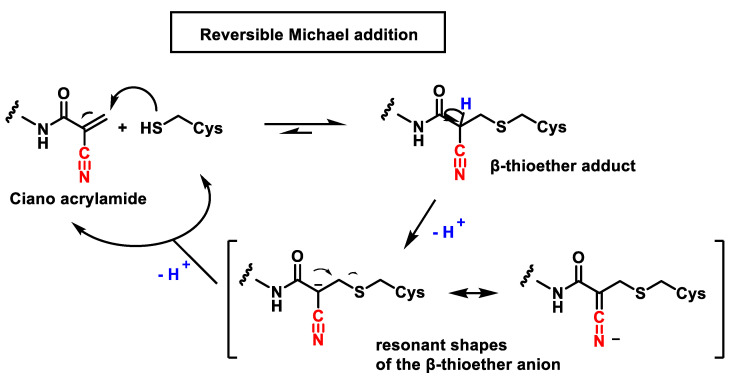
Mechanism of reversible inhibition by conjugate addition to cyano acrylamides.

Serafimova et al., 2012 were the first to propose a reversible covalent inhibitor of the synthetic p90 ribosomal protein S6 kinase RSK2 by introducing an electron-accepting cyan group at the α-position of the acrylamide [40]. The concept of reversible conjugative addition was introduced by Taunton and co-workers who found that by introducing an additional electron-accepting moiety (e.g., a cyano group) at the α-position of an acrylamide, the acidity of the α-proton of the β-thioether adduct increases favoring the reverse reaction [40,41,42] (Figure 6).

The introduction of a cyano group at the α-position of acrylamide can not only increase its electrophilicity and reactivity with cysteine residues but also greatly enhance the acidity of α-H so that the reverse Michael addition reaction can occur biologically in vivo. By considering the steric hindrance of the β-group, the rate of α-H elimination can be regulated, thus modulating the rate of the reverse Michael addition reaction [39].

The applicability of this concept was demonstrated by the design of selective and nearly irreversible inhibitors of the ribosomal s6 kinase (RSK) family and Bruton’s tyrosine kinase (BTK) inhibitors. Reversible covalent inhibitors are not permanently bound and can be released from non-target proteins, reducing the potential for undesirable immune system activation and off-target toxicity [43,44].

**Figure 7:**
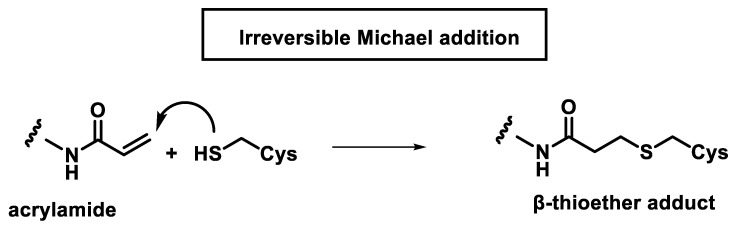
Mechanism of irreversible inhibition by conjugate addition to acrylamides.

Irreversible covalent bond formation occurs between acrylamides and thiols generating kinetically stable adducts (Figure 7).

### 2.3. Reversible and Irreversible Covalent Binding to Proteins

Covalent binding to proteins is a crucial concept in biochemistry and refers to the formation of a chemical bond between a molecule and a specific amino acid side chain within a protein [7,45]. This bond formation significantly alters the protein’s structure and function and has numerous applications in various fields including the following:

- Drug discovery and development, by covalently binding to specific target proteins, thereby inhibiting their function or altering their activity.
- Inhibition of enzymes by binding to and inactivating them, thereby interfering with the regulation of cellular processes.
- Protein engineering, inducing covalent modifications in proteins modifies their stability, function or other properties.

Covalent inhibitors of proteins can be classified as reversible and irreversible. Irreversible covalent inhibitors are small molecule drugs that covalently bind irreversibly to their target proteins and this mechanism has several advantages over conventional reversible inhibitors, including increased duration of action, less-frequent drug dosing, reduced pharmacokinetic sensitivity and the potential to target intractable shallow binding sites. However, the key challenges of irreversible covalent drugs are their potential for off-target toxicities and immunogenicity risks [46].

Reversible covalent inhibitors in covalent drugs would lead to less off-target toxicity by forming reversible adducts with off-target proteins and thus reducing the risk of idiosyncratic toxicities caused by the permanent modification of proteins [46]. This approach has garnered much attention in drug discovery in the last two decades and has now become a common modality for protein target inhibition. The lifetime of the covalent, inhibited complex is governed by the reverse reaction back to the non-covalent complex. For completely irreversible covalent inhibitors, the reverse reaction is effectively zero and so their concentration and time-dependent target inhibition are solely governed by the binding affinity and rate of covalent bond formation [47].

The incorporation of drugs that bind covalently and reversibly to proteins would lead to lower toxicity by forming reversible adducts and thus reduce the risk of idiosyncratic toxicities caused by permanent modification of proteins, leading to higher levels of potential haptens [48].

## 3. Transcription Factors That May Be Potential Targets of Michael Acceptor Compounds in Neoplastic Metabolism

MA compounds develop their biological activity through the presence of electrophilic groups in their structure, which influence gene expression at various levels:

- They can interact with transcription factors, altering their ability to activate or repress the transcription of certain genes [49].
- They can affect the stability of mRNA and thus influence the production of certain proteins. Changes in mRNA stability have a significant impact on protein production in cells [50].
- They can affect mRNA translation into proteins [51]. An example of this is some MA compounds that inhibit the main protease of SARS-CoV-2, which is important for viral replication [52].

These modulations of gene expression can lead to an increased production of antioxidant enzymes and reduced inflammatory proteins [13]. The target proteins of MA compounds include the peroxisome proliferator-activated receptor γ (PPAR-γ), the pro-inflammatory and tumorigenic NF-κB signaling pathway, the transcription factor STAT3, nuclear exportin-1 (XPO1) and the c-Myc oncoprotein.

### 3.1. Transcription Factor NF-κB

The family of NF-κB transcription factors, which includes the proteins p65, p50, p52, RelB and c-Rel [53], plays a central role in the regulation of genes that control both innate and adaptive immune responses. Activation of NF-κB is usually initiated by extracellular ligands that bind to specific cell surface receptors, including hormones, neurotransmitters, cytokines, growth factors and nutrients [54]. After activation, NF-κB translocates to the nucleus, binds to DNA response elements and initiates the transcription of target genes, including the pro-inflammatory response. It is a primary “fast-acting” transcription factor, with a critical role in pathological conditions [55]. The canonical activation of NF-κB metabolism is triggered by oxidative stress and pro-inflammatory cytokines [56].

The IKK complex plays a central role in the inactivation of NF-κB by phosphorylating the inhibitory IκBα protein, leading to its dissociation from NF-κB. This process allows NF-κB to migrate into the nucleus and activate the expression of genes, including those with anti-apoptotic functions [57]. Abnormally high NF-κB activity in many cancers, due to genetic mutations, chronic inflammation and exposure to carcinogens, contributes to cancer development and progression through mechanisms such as promoting cell proliferation, inhibiting apoptosis, facilitating angiogenesis and triggering epithelial-to-mesenchymal transition (EMT) [58].

MA compounds regulate the NF-κB signaling pathway by targeting nucleophilic residues of disease-related proteins and provide therapeutic effects with low toxicity in various diseases. MAs reduce IKK activity and NF-κB activation via antioxidant properties, due to the alteration of the NF-κB subunit composition and disruption of upstream signaling pathways [13,59,60,61]. In sum, targeting NF-κB is a promising strategy for cancer treatment, and current research is focused on developing inhibitors.

### 3.2. Transcription Factor PPAR-γ

The transcription factor Peroxisome Proliferator-Activated Receptor gamma (PPAR-γ) is involved in the regulation of lipid homeostasis, inflammatory signaling and adipocyte differentiation. It belongs to the nuclear hormone receptor superfamily and comprises three isoforms: PPAR-α, PPAR-γ and PPAR-β/δ. Activation of PPAR-γ leads to insulin sensitization, and increased glucose metabolism and is essential for the differentiation and proliferation of adipocytes, facilitating the uptake and storage of fatty acids [62] and plays an important role in various diseases:

- PPAR-γ agonists can suppress inflammatory responses in psoriatic skin lesions and attenuate associated comorbidities [63].
- Neurodegenerative diseases, signaling networks, insulin sensitivity, glucose homeostasis, fatty acid oxidation, immune responses, redox balance, cardiovascular integrity and cell fate depend on the PPAR-γ factor [64]. PPAR-γ agonists can reduce amyloid and tau pathologies, and neuroinflammation and improve memory impairment in models of Alzheimer’s disease [65].
- Suppression of PPAR-γ under disease conditions exacerbates inflammatory and fibrogenic factors, contributing to kidney damage [66].
- PPAR-γ is associated with susceptibility to neuronal damage in models of brain disease [67].

PPAR-γ plays a critical role in various cancers by regulating cell proliferation in the colon, breast, prostate and urinary bladder, and dysregulation of PPAR-γ signaling is associated with tumor development [68]. PPAR-γ inhibits cell proliferation, induces cell cycle termination and apoptosis in various cancer cell types, promotes intercellular adhesion and mitigates the inflammatory state of the tumor microenvironment [69].

PPAR-γ is a target for integrative therapies that simultaneously act on tumors and their microenvironment [70] because it regulates gene expression in multiple signaling pathways, including B-cell lymphoma 2 (BCL-2), nuclear factor kappa light-chain enhancer of activated B cells (NF-κB), tumor suppressor p53 (p53) and cyclooxygenase-2 (COX-2), which play a role in various diseases, including cancer [70].

### 3.3. Transcription Factor STAT3

STAT3, also known as Signal Transducer and Activator of Transcription 3, is a key protein involved in various cellular processes, including cell proliferation, apoptosis, cell differentiation and inflammation [71]. Its activation is triggered by various signals, such as cytokines, growth factors and hormones. Upon activation, STAT3 is phosphorylated at two residues, tyrosine 705 and serine 727, allowing it to dimerize and translocate to the nucleus. In the nucleus, STAT3 binds to specific DNA sequences and initiates the transcription of genes involved in cellular processes such as proliferation and growth factor signaling [72]. It is also involved in inflammation, where cytokines such as interleukin-1 (IL-1) and IL-6 activate STAT3, leading to the expression of genes that promote inflammation [73]. The role of STAT3 in cancer is complex:

- STAT3 contributes to metastasis and drug resistance; its hyperactivation is associated with a poor clinical prognosis in most human cancers [74].
- STAT3 is observed in both cancer and non-cancer cells in the tumor microenvironment, inhibits the expression of immune activation and promotes immunosuppressive factors, thus contributing to tumor progression [75].
- The interplay between STAT3 and non-coding RNAs has attracted attention and highlighted its regulatory role in gene expression networks [76].

Drugs targeting STAT3 activity are being explored as potential cancer treatments. These therapies aim to selectively block specific aspects of cell or tumor biology associated with STAT3, offering a tailored approach compared to conventional chemotherapy. By selectively inhibiting STAT3, these drugs have the potential to cause less damage to healthy cells while effectively targeting cancer cells. They can be administered alone or in combination with standard chemotherapy to increase the effectiveness of the treatment [77].

### 3.4. The Nuclear Export Receptor Exportin-1

The XPO1 nuclear export receptor exportin-1 (or chromosomal region maintenance 1 CRM1), plays a crucial role in mediating the nuclear export of proteins containing a leucine-rich nuclear export signal (NES) into the cytoplasm. This protein is located in the nuclear membrane and facilitates the transport of approximately 220 proteins from the nucleus and exports various proteins from the nucleus, including nuclear receptors, transcription factors, proteins that regulate the cell cycle and stress response proteins [78].

Its role is crucial for the regulation of gene expression and cellular function [79]. Disruption of this process can lead to various diseases and research on XPO1 has intensified in recent years, with a focus on the development of new cancer therapies. Its inhibition can induce apoptosis in cancer cells, so it is an attractive target for cancer treatment [80].

XPO1 is upregulated in many cancers and is considered an important target in hematologic malignancies, tumor resistance, inflammation, neurodegeneration and viral infections. Pharmacological inhibition of XPO1 is of great therapeutic interest [81,82,83,84,85,86,87,88,89,90,91,92,93,94,95,96,97,98,99]. Promising results have been observed with inhibitors such as leptomycin B and selective inhibitors of nuclear export (SINEs). These inhibitors interact with XPO1 via a covalent interaction with Cys528, which is in the NES binding cleft of XPO1. In particular, the SINE inhibitors cause a reduction in XPO1 protein levels via the proteasomal pathway, offering a potential therapeutic benefit in various diseases, especially cancer [100].

### 3.5. Oncoprotein c-Myc

The Myc family of proto-oncogenes consists of several members, including L-myc, N-myc and c-myc, which are found in normal cells. These genes encode proteins that are in the cell nucleus, bind to DNA and facilitate transcription, thereby regulating the activity of other genes [101]. Proto-oncogenes are genes whose activation into oncogenes can drive cancer development. When over-activated in normal cells, they can cause uncontrolled division and proliferation, leading to cancer. Among the members of the Myc family, c-Myc is the best studied and is expressed in cells with a high proliferation rate [102].

Alterations in c-Myc are frequently observed in many solid tumors, leukemias and lymphomas. These alterations include amplifications, translocations, mutations and chromosomal rearrangements that affect the c-Myc gene locus and lead to the dysregulation of c-Myc expression in various human neoplasms. Activation of the c-Myc proto-oncogene often occurs through amplification mechanisms leading to its overexpression. This overexpression is found in various cancers such as lung cancer, breast cancer, neuroblastoma, glioblastoma, gastric cancer, colon cancer, Burkitt’s lymphoma and others [103].

Loss of upregulation of c-Myc is common in certain cancers such as breast, lung, ovarian and prostate cancer, while loss of its regulation is common in colon cancer, gynecologic tumors and melanoma [104]. The c-Myc protein functions as a transcription factor that binds DNA non-specifically, but also recognizes specific nuclear sequences such as 5′-CAC[GA]TG-3′. It activates the transcription of growth-related genes, including the promotion of VEGFA production, which stimulates sprouting angiogenesis [105].

The c-Myc protein is a nuclear phosphoprotein consisting of 439 amino acids with several conserved structural sequences, with two main domains: a dimerization motif, necessary for the formation of heterodimers with other proteins such as Max and binding to DNA at specific E-box sequences, and a transactivation domain, crucial for its oncogenic activity. The c-Myc protein has a short half-life of about 20 minutes, whereas the half-life of the Max protein is more than 24 h. This property makes c-Myc the limiting component of the Myc-Max complex [106,107,108,109,110].

## 4. Michael Acceptors in Current Chemotherapy Treatments

In cancer chemotherapy, MAs are used to target specific enzymes and proteins that are crucial for the growth and survival of cancer cells. Their α,β-unsaturated carbonyl group gives them a high reactivity, allowing them to form covalent bonds with nucleophilic residues on proteins. This reactivity is used to selectively attack enzymes and proteins involved in carcinogenesis.

MAs can undergo Michael addition reactions with cysteine residues, a key mechanism used in cancer therapy. This reaction leads to post-translational modifications of selected regulatory proteins that alter their function within cellular signaling pathways or biosynthetic processes. By modifying these proteins, MAs disrupt essential cellular functions that are critical for the proliferation, survival and metastasis of cancer cells. Consequently, MAs represent a promising strategy in cancer therapy, offering the potential for improved efficacy and reduced toxicity compared to conventional chemotherapeutics.

### 4.1. Tyrosine Kinase Inhibitors (TKIs)

Tyrosine kinases (TKs) are enzymes that are crucial for the activation of numerous proteins via signal transduction cascades. Phosphorylation, a crucial step in this process, is inhibited by tyrosine kinase inhibitors (TKIs). These TKIs are targeted therapies that are widely used to treat various types of cancer [111].

Receptor tyrosine kinases (RTKs) are a specific subgroup of tyrosine kinases responsible for promoting cell growth and proliferation. Dysregulation of RTK expression leads to abnormal cell growth and contributes to tumorigenesis, angiogenesis and metastasis. Consequently, targeting RTK is a promising approach to prevent cancer progression [112].

**Figure 8:**
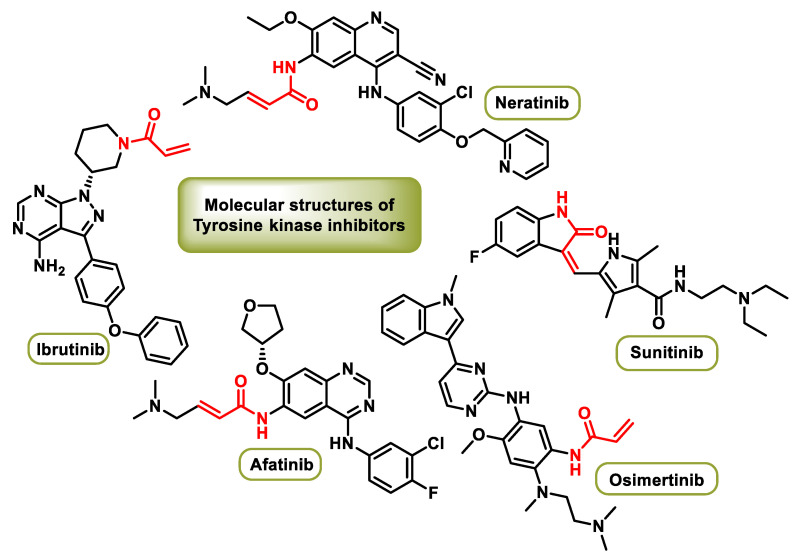
Molecular structures of tyrosine kinase inhibitors.

TKIs exert their therapeutic effect by selectively inhibiting the activity of these tyrosine kinases, thereby disrupting critical signaling pathways involved in carcinogenesis. By interfering with these molecular processes, TKIs offer a precise and tailored approach to cancer treatment and can potentially minimize the negative effects on healthy tissue [113]. Figure 8 shows several molecular structures relevant to these targeted therapies.

Afatinib is used for the treatment of non-small cell lung cancer (NSCLC). It is primarily prescribed for cases of NSCLC characterized by mutations in the epidermal growth factor receptor (EGFR) gene. By targeting EGFR mutations, afatinib interferes with signaling pathways that are critical for cancer cell proliferation and survival, providing a tailored therapeutic approach for NSCLC patients with specific genetic alterations [114].

Neratinib is implicated in the treatment of breast cancer and is specifically recommended for extended adjuvant therapy in adults with hormone receptor-positive HER2-overexpressed/amplified early breast cancer who have received prior adjuvant therapy with trastuzumab within one year. Neratinib inhibits HER2 and interrupts the signaling pathways responsible for breast cancer progression, providing a targeted therapeutic option for this subtype of breast cancer [115].

Sunitinib, a multicenter RTK inhibitor, received FDA approval for the treatment of renal cell carcinoma (RCC) and imatinib-resistant gastrointestinal stromal tumors (GISTs). As the first cancer drug to be approved for two different indications, sunitinib demonstrates efficacy in targeting multiple signaling pathways involved in tumor growth and angiogenesis, providing a versatile therapeutic approach for RCC and GIST patients [116].

Osimertinib, which is used in the treatment of non-small cell lung cancer with specific mutations, selectively inhibits the epidermal growth factor receptor (EGFR) tyrosine kinase at its Cys797 residues in the ATP-responsive site. By targeting EGFR mutations, osimertinib disrupts downstream signaling pathways that are important for cancer cell survival, providing a targeted therapy option for NSCLC patients with specific genetic alterations [117].

Ibrutinib, a Bruton’s tyrosine kinase (BTK) inhibitor, serves as an antineoplastic drug that targets B-cell cancers. Ibrutinib, which is FDA-approved for the treatment of Waldenstrom’s macroglobulinemia, a form of non-Hodgkin’s lymphoma, inactivates BTK by covalently binding cysteine-481 near the ATP-binding domain [118]. Through this mechanism, ibrutinib disrupts the B-cell signaling pathways involved in carcinogenesis, providing a targeted therapeutic option for B-cell malignancies.

### 4.2. Cyclin-Dependent Kinases (CDKs) Inhibitors

Cyclin-dependent kinases (CDKs) are a family of protein kinases that have been shown to be important regulators of cell cycle progression [119]. The activity of CDKs is controlled by their association with cyclins and by the action of CDK inhibitors (CDKIs). CDKIs inhibit the function of CDKs and they are used to treat cancers by preventing the overproliferation of cancer cells. While there are multiple cyclin/CDK complexes regulating the cell cycle, CDKIs targeting CDK4/6 have been the most successful and four CDK4/6 inhibitors have been FDA approved [120].

**Figure 9:**
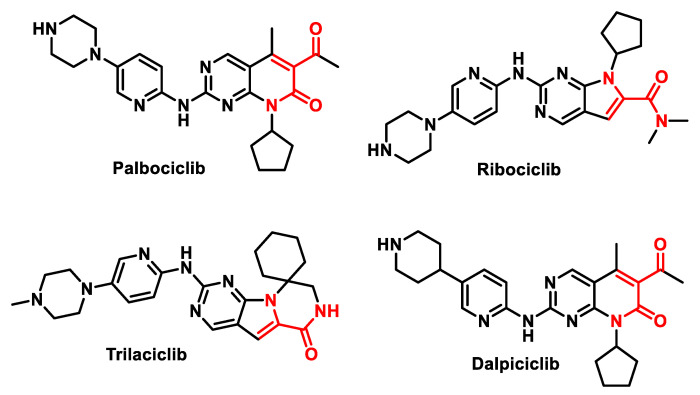
Molecular structures of CDK inhibitors.

In recent years, CDKIs have emerged as promising therapeutic agents in oncology. For example, CDK4/6 inhibitors such as palbociclib, ribociclib, trilaciclib and dalpiciclib have become standard-of-care therapy for several cancers [121] (Figure 9).

Malumbres et al., 2008 categorized CDKIs based on their target specificity: broad CDKIs, specific CDKIs and multiple target inhibitors. The current FDA-approved drugs are all CDK4/6 inhibitors targeting CDK4 and CDK6, two enzymes that control the cell cycle checkpoint transition checkpoint from the G1 to the S phase of the cell cycle [122].

### 4.3. Aurora Kinase (AURK) Inhibitors

Aurora kinases (AURKs) are serine/threonine kinases essential for cell proliferation, as they help the dividing cell to distribute its genetic material to daughter cells, playing a crucial role in cell division by controlling chromatid segregation. The genetic overexpression or amplification of AURKs has been observed in several cancers, so their inhibition could enhance the effect of chemotherapies [123].

**Figure 10:**
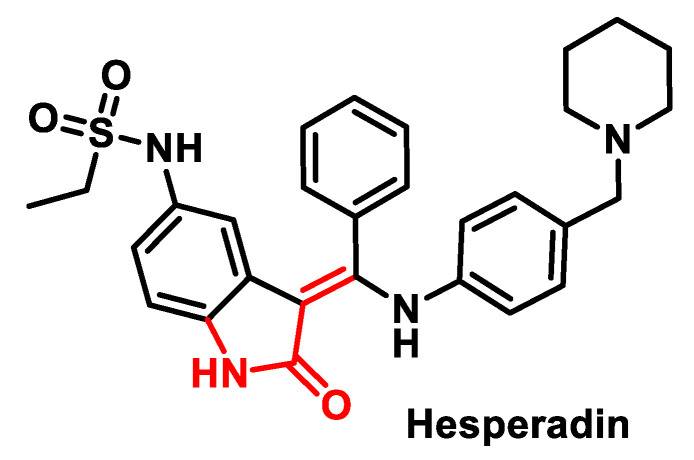
Molecular structure of Hesperadin.

Inhibiting cancer growth by developing drugs that target enzymes that regulate cell mitosis is a promising new approach [124]. Three AURK inhibitors have been described so far: ZM447439 [125], Hesperadin [126] and VX-680. Hesperadin, (Figure 10), potently inhibits Aurora B with IC50 of 250 nM in a cell-free assay. It markedly reduces the activity of AMPK, Lck, MKK1, MAPKAP-K1, CHK1 and PHK while it does not inhibit MKK1 activity in vivo.

### 4.4. Bruton’s Tyrosine Kinase (BTK) Inhibitors

**Figure 11:**
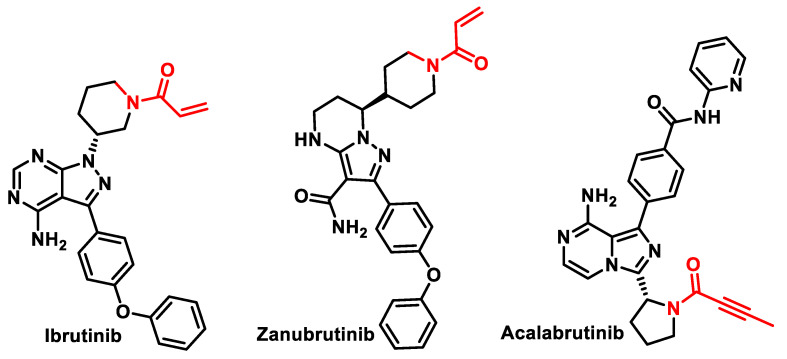
Molecular structures of BTK inhibitors.

Bruton’s tyrosine kinase (BTK) is a key component of the B-cell receptor (BCR) signaling pathway. BTK is a regulatory protein for cell proliferation (growth) and cell survival in several B-cell lymphomas. BTK inhibitors (Figure 11) are a type of drug that works to treat cancers caused by defective B cells, such as chronic lymphocytic leukemia, B-cell lymphomas, and Waldenström macroglobulinemia. BTK inhibitors act by interfering with the B-cell receptor signaling pathway. When the B-cell receptor signaling pathway goes haywire, B-cells can reproduce at an uncontrolled rate and cause lymphoma. By disrupting this pathway, the abnormal B cells will not divide.

### 4.5. Nitro Fatty Acids (NO_2_-FAs)

**Figure 12:**
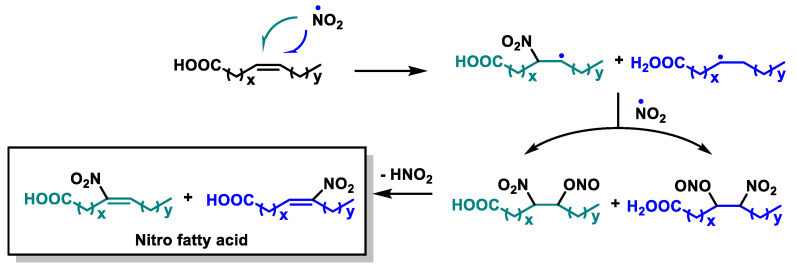
Endogenous nitration of UFAs.

Nitro-unsaturated fatty acids (NO_2_-FAs) represent a class of molecules that can be generated endogenously by the reaction of unsaturated fatty acids (UFAs) with secondary nitrogen dioxide (NO_2_) species and nitrite anions [127] (Figure 12).

The NO_2_-FAs moiety, characterized by a nitroalkene structure, acts as a potent MA that can react with thiol-containing residues in biologically relevant proteins. This reactivity enhances the therapeutic potential of NO_2_-FAs [128].

In addition to their reactivity with proteins, NO_2_ FAs exhibit broader biological effects. They generally inhibit the activity of nuclear factor κB (NF-κB), a transcription factor involved in inflammation, while simultaneously activating NRF2, which initiates an antioxidant signaling pathway. This dual action makes NO_2_-FAs promising candidates for therapeutic intervention in diseases associated with oxidative stress and inflammation [129].

**Figure 13:**
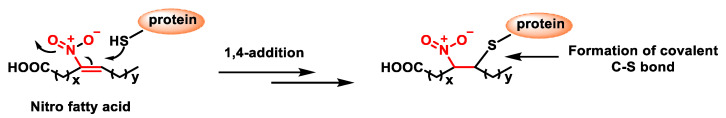
NO_2_-FAs reaction with the thiol group of biologically relevant proteins.

NO_2_-FAs can be synthesized both endogenously in the body and exogenously in the laboratory. Stepwise synthesis requires specific precursor compounds and can yield NO_2_-FAs with precise positional modifications (Figure 13).

In terms of their molecular targets, NO_2_-FAs can modulate the activity of various proteins involved in inflammatory and tumorigenic processes. These target proteins include peroxisome proliferator-activated receptor γ (PPAR-γ) [61], which regulates lipid metabolism and inflammation, the pro-inflammatory and tumorigenic NF-κB signaling pathway [130], the pro-inflammatory 5-lipoxygenases (5-LO) biosynthetic pathway [131] and soluble epoxide hydrolase (sEH), an important regulator of vascular tone [132]. By interacting with these proteins, NO_2_-FAs exert diverse effects on cellular signaling and physiology, offering potential therapeutic opportunities for diseases characterized by inflammation and impaired lipid metabolism.

### 4.6. Anthracycline Family of Chemotherapy Drugs

Anthraquinones and their derivatives are widely used in medicine, particularly in the treatment of various types of cancer [133]. Among these derivatives, anthracenediones and anthracyclines stand out as chemotherapeutic agents that are widely used in oncological practice. Anthracyclines, which are derived from Streptomyces bacteria, have been shown to be effective against a wide range of cancers, including leukemias, lymphomas, breast cancer, gastric cancer, uterine cancer, ovarian cancer, bladder cancer and lung cancer.

The discovery of anthracyclines was a major milestone in cancer therapy, with daunorubicin being the first member of this class to be identified. Daunorubicin, which is naturally produced by *Streptomyces peucetius* [134], paved the way for the development of subsequent clinically important anthracyclines. The best-known anthracyclines used in clinical practice include the following:

- Doxorubicin, which is used in the treatment of breast cancer, lung cancer, ovarian cancer, liver cancer, thyroid cancer, leukemias and lymphomas [135].
- Daunorubicin, which is used in the treatment of acute myeloid leukemia (AML), acute lymphoblastic leukemia (ALL), chronic myeloid leukemia (CML) and Kaposi’s sarcoma.
- Epirubicin, which is used for breast cancer, ovarian cancer, stomach cancer, lung cancer and lymphoma.
- Idarubicin, which is prescribed for acute myeloid leukemia.

**Figure 14:**
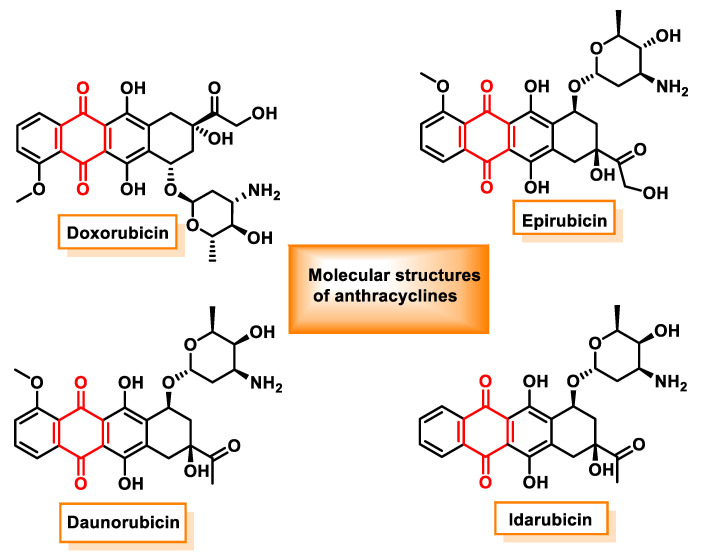
Molecular structures of anthracyclines.

These anthracyclines exert their therapeutic effect primarily by intercalating with DNA, thereby interfering with DNA metabolism and RNA production in cancer cells. However, their clinical utility is often limited by significant adverse effects, particularly cardiotoxicity, which may compromise their long-term use and overall efficacy. Despite these limitations, anthracyclines remain an integral part of many cancer treatment regimens due to their potent antitumor activity against a variety of malignancies (Figure 14).

### 4.7. Family of Selective Inhibitors of Nuclear Export (SINEs)

Inhibiting XPO1 has proven to be a promising strategy in cancer therapy, as it plays an important role in the transport of proteins from the cell nucleus into the cytoplasm. Leptomycin B, a potent inhibitor of XPO1, was the first candidate. However, its high toxicity to normal cells limited its clinical utility, so alternative compounds with similar mechanisms of action were explored. These compounds aim to covalently bind to the Cys528 residue in the binding groove of the nuclear export signal (NES) of XPO1, thereby blocking its function [100].

Selective inhibitors of nuclear export (SINEs) are a class of drugs designed to block the activity of XPO1, leading to cell cycle arrest and apoptosis. They are of great interest as potential anti-cancer drugs and several of them are in development. Selinexor, a SINE, has been approved for the treatment of multiple myeloma in refractory cases, highlighting the clinical potential of this class of drugs. SINEs exert their anti-cancer effects by restoring normal nuclear transport of various proteins, including tumor suppressors and oncogenes, thereby promoting apoptosis in malignant cells [136].

**Figure 15:**
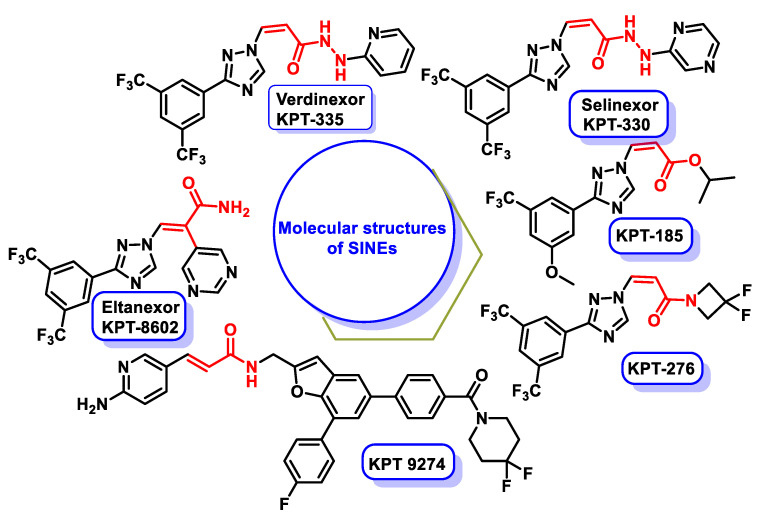
Molecular structures of SINEs.

Preclinical studies have demonstrated the efficacy of SINEs in various animal models of cancer, including pancreatic cancer, breast cancer, non-small cell lung cancer, lymphomas and acute and chronic leukemias [137]. In addition, early-stage human clinical trials have shown promising results in a spectrum of advanced or refractory solid tumors, including non-Hodgkin’s lymphoma, colorectal cancer, head and neck cancer, melanoma, ovarian cancer and prostate cancer [138]. The molecular structures of SINEs are shown in Figure 15 and illustrate the diversity of compounds within this class that have the potential for cancer therapy.

## 5. Natural Michael Acceptors in Cancer Prevention and Treatment

The compounds discussed in this section are secondary metabolites from medicinal plants known for their anti-cancer properties. These compounds mainly belong to classes such as alkaloids, terpenes, polyketides and polyphenols. Nature has given us various compounds with strong anti-cancer effects. Some examples include well-known active substances such as vincristine and taxol, which are currently used as chemotherapeutic agents [139].

Despite the existence of established anti-cancer drugs, the search for new therapeutics has been a long-standing challenge for science. Research into naturally occurring compounds offers a promising avenue in this search [140]. Many natural compounds, some of which are even found in the human diet, possess anti-cancer properties and exert their effects through reactivity facilitated by an MA group.

These natural compounds have a wide variety of chemical structures and mechanisms of action and act on different molecular pathways involved in cancer development and progression. Furthermore, their origin from plant sources underlines the potential of natural products as valuable reservoirs for anti-cancer drugs.

### 5.1. Alkaloids

**Figure 16:**
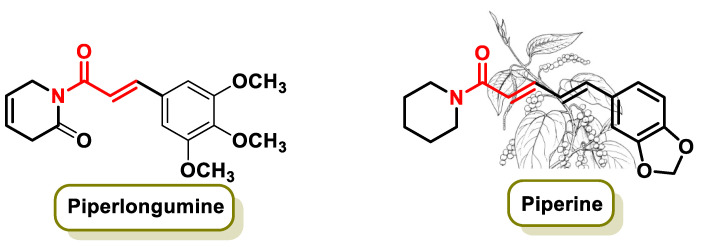
Piperlongumine and piperine molecular structure.

Alkaloids represent an important category of secondary metabolites known for their potential in cancer prevention and therapy. Recent research by Chen et al. 2020 demonstrated the synergistic inhibitory effect of two alkaloids, Piperine and Piperlongumine (Figure 16), on breast cancer cells by effectively blocking STAT3 activation [141]. The alkaloids Piperlongumine and Piperine are derived from Indian pepper and black pepper (*Piper nigrum*), respectively (Figure 16). These compounds are mainly found in the outer layer of the fruit and are characterized by a spicy or pungent sensation together with a bitter taste.

The results of the study highlight the therapeutic potential of these alkaloids in fighting breast cancer by targeting the activation of STAT3, a crucial signaling pathway that plays a role in cancer development [141]. This research underscores the importance of alkaloids as promising candidates for cancer treatment and highlights the importance of researching natural compounds for their anti-cancer properties.

In 2012, a remarkable research study showed that piperine can prevent the formation of new fat cells and lower blood lipid levels by inhibiting the genes responsible for the formation of new fat cells [142]. Piperine, which is extracted from black pepper, has several pleiotropic properties, including antioxidant, anti-cancer, anti-inflammatory, anti-hypertensive, hepatoprotective and neuroprotective activities [143]. It is also associated with influencing gastrointestinal disorders and drug-metabolizing enzymes. It improves the bioavailability of various drugs and offers therapeutic benefits for people with diabetes, obesity, metabolic syndrome and hypertension [143]. Piperine exerts its effects by inhibiting cytochrome P450 3A4 (CYP3A4), a key enzyme involved in initial drug metabolism and the P-glycoprotein transporter, which facilitates the rapid efflux of drugs from the body [144].

In addition, piperine has been found to have chemopreventive properties, interfering with the development and progression of cancer [145]. It stimulates the immunomodulatory activities of mononuclear cells by producing anti-inflammatory cytokines and inhibiting pro-inflammatory activities, thereby exerting a pronounced anti-cancer effect [146]. Piperine affects cancer cells via several mechanisms, including the modulation of redox homeostasis, inhibition of cancer stem cell (CSC) self-renewal and the modulation of ER stress and autophagy. It can also alter the activity of numerous enzymes and transcription factors to inhibit invasion, metastasis and angiogenesis [147].

**Figure 17:**
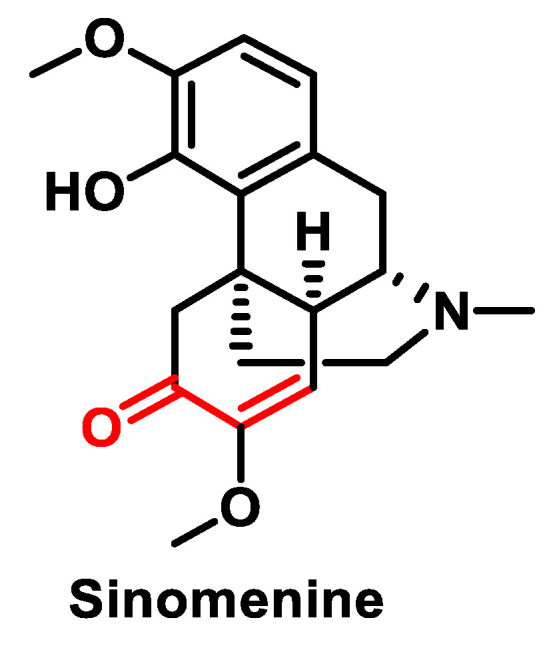
Sinomenine molecular structure.

Another natural compound with therapeutic potential is Sinomenine (Figure 17), which is extracted from the medicinal plant *Sinomenium acutum*. Pharmacological studies have shown that Sinomenine has therapeutic effects on various diseases such as rheumatoid arthritis, pain, atherosclerosis and cancer [148,149]. This substance represents a promising avenue for further exploration in the field of medical research and treatment strategies.

### 5.2. Terpenes and Terpenoids

Terpenes and terpenoids form a class of secondary metabolites synthesized by plants and insects. While terpenes are hydrocarbons, terpenoids are their oxygenated derivatives. This diverse group includes monoterpenes, sesquiterpenes, diterpenes, triterpenes and tetraterpenes, each with unique chemical structures and biological activities. Several terpenes have shown promising potential in the context of cancer therapy [150].

#### 5.2.1. Sesquiterpenes

**Figure 18:**
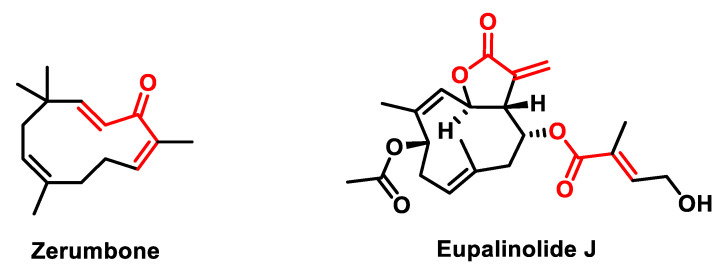
Zerumbone and Eupalinolide J molecular structure.

Sesquiterpenes, also known as sesquiterpenoids, are a class of terpenes consisting of 15 carbon atoms. Zerumbone and Eupalinolide J (Figure 18) represented by the chemical structure (2,6,9,9-tetramethyl-[2 E, 6 E, 10 E]-cycloundeca-2,6,10-trien-1-one), is an example of such compounds. It is a monocyclic sesquiterpene characterized by three double bonds, two of which are conjugated, together with an isolated double bond and a conjugated carbonyl group forming an 11-membered ring structure [151].

Zerumbone is mainly found in the essential oil of *Zingiber zerumbet* [152], commonly known as pinecone ginger. It has attracted considerable attention due to its potential therapeutic effects, particularly in the treatment of cancer. Studies have shown that Zerumbone triggers apoptosis or programmed cell death. This is the primary mechanism underlying its antiproliferative effect in various tumor cell lines [153].

Numerous preclinical studies have shown the promising efficacy of Zerumbone in the treatment of various types of cancer, including colon, breast, cervical and liver cancer. It has been shown to inhibit the proliferation of cancer cells while exhibiting selective cytotoxicity against cancer cells compared to normal cells. Zerumbone exerts its proliferation-inhibiting effect by targeting key proteins involved in the regulation of cell growth [154,155,156].

In addition, Zerumbone exhibits anti-inflammatory properties that contribute to its potential as a therapeutic agent against cancer and other inflammatory diseases. Its ability to inhibit cell growth has been demonstrated in a spectrum of cancer cell lines including blood, skin, breast, liver, lung and colorectal cancers [157,158,159,160].

Overall, Zerumbone is a promising natural compound with anti-cancer properties that represents a potential avenue for the development of novel cancer therapeutics [161].

Eupalinolide J (EJ) is a sesquiterpene lactone isolated from *Eupatorium lindleyanum* DC with promising anti-cancer activity via the STAT3 and Akt signaling pathways, inhibiting cancer cell metastasis both in vitro and in vivo [162]. It has also been shown to have antiproliferative effects in human prostate cancer cells, inducing cell apoptosis and cell cycle arrest. EJ showed marked anti-proliferative activity in PC-3 and DU-145 cells in a dose- and time-dependent manner. The activation of caspase-3 and caspase-9 was visibly observed. Furthermore, the expression levels of γH2AX, p-Chk1 and p-Chk2 were significantly upregulated, suggesting the induction of DNA damage responses in EJ-treated prostate cancer cells. The above results indicated that EJ exhibited effective anti-cancer activity in vitro. It could be a promising candidate agent for the clinical treatment of prostate cancer [163].

#### 5.2.2. Diterpenoids

Diterpenoids are a diverse group of compounds characterized by a C20 carbon skeleton consisting of four isoprene units. These natural products are formed by the condensation of a dimethylallyl diphosphate (DMAPP) unit and three isopentenyl diphosphate (IPP) units via the Mevalonic acid pathway [164].

**Figure 19:**
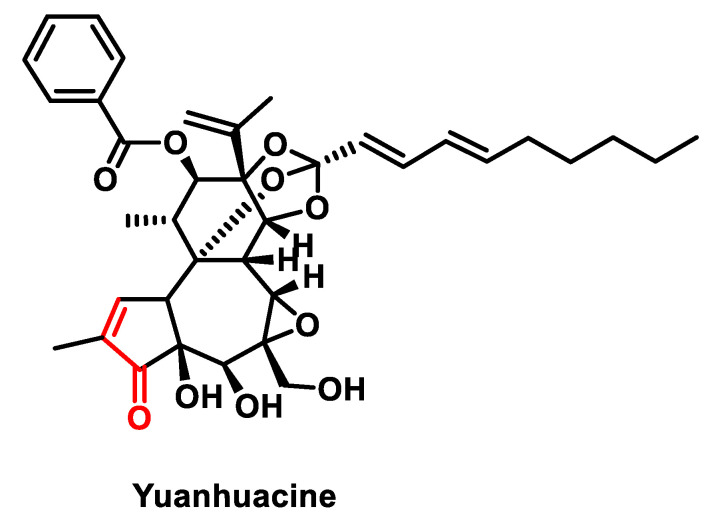
Yuanhuacine molecular structure.

Yuanhuacin, also known as Gnidilatidin, (Figure 19), is a remarkable diterpenoid isolated from the flowers of *Daphne genkwa*. This compound has attracted attention due to its significant anti-tumor activity, particularly in the context of non-small cell lung cancer (NSCLC) [165].

Studies have shown that Yuanhuacin exerts its anti-tumor effects by regulating signaling pathways involved in cellular metabolism and proliferation. It has been shown to modulate the adenosine monophosphate-activated protein kinase (AMPK) and mTOR (mammalian target of rapamycin) signaling pathways. The AMPK is an important regulator of cellular energy homeostasis, while the mTOR plays a central role in the control of cell growth and metabolism [166].

The discovery of Yuanhuacin and its anti-tumor properties underscores the importance of diterpenoids as promising candidates for the development of new cancer therapeutics [165]. Further research into the molecular mechanisms underlying its activity and its potential clinical applications is warranted to fully exploit its therapeutic potential in cancer treatment.

#### 5.2.3. Triterpenoids or Steroids

Triterpenic acids represent a class of natural secondary metabolites that have the remarkable ability to induce apoptosis, a process that is critical for controlling the growth of cancer cells, while exhibiting low cytotoxicity. To enhance their anti-cancer activity, an effective strategy is to incorporate a functional MA group into their molecular structure, as this modification often confers increased cytotoxicity to the compounds [167].

In a 2015 study by Heller et al., the researchers synthesized and evaluated several MA-substituted compounds derived from Glycyrrhetinic acid, Ursolic acid, Oleanolic acid and Platanoic acid. When investigating the influence of an MA moiety on the cytotoxicity of various human cancer cell lines, they observed a significant enhancement of the cytotoxic activity of these triterpenic acid derivatives [168].

Triterpenoids, i.e., triterpenes containing heteroatoms, typically oxygen, are often equated with the term triterpenes. This class of compounds encompasses a broad spectrum of cyclic structures, including more than 4000 known members, including both free triterpenoids and saponins with triterpenoid aglycones. Notable subclasses such as ursans, oleanans, lupans, dammarans and euphans exhibit diverse biological activities, including anti-inflammatory, anti-cancer, antidiabetic, hepatoprotective, antimicrobial, antifungal, analgesic, immunomodulatory and cardiotonic properties [169].

**Figure 20:**
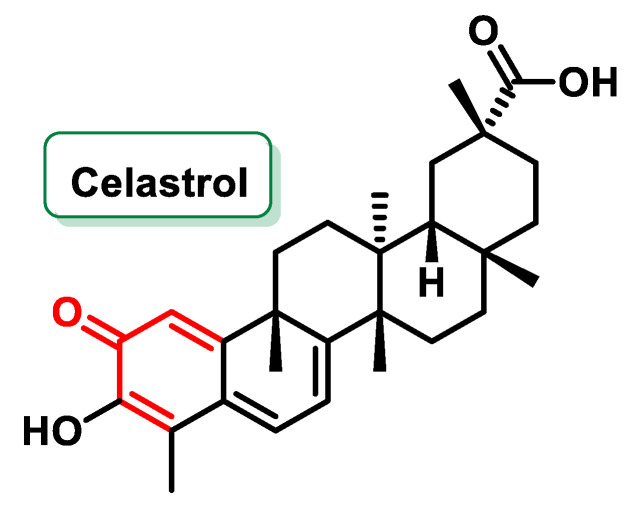
Celastrol molecular structure.

Celastrol (Figure 20) is a pentacyclic triterpenoid of the quinone methylide family isolated from the root extracts of *Tripterygium wilfordii* and *Celastrus regelii*. Celastrol exhibits a wide range of pharmacological activities, including antioxidant, anti-inflammatory, anti-cancer and insecticidal properties. Its ability to modulate various cellular signaling pathways underscores its potential as a promising candidate for therapeutic intervention in cancer and other diseases [170].

In 2015, Liu et al. discovered that Celastrol has potent anti-obesity properties by acting as a leptin sensitizer. Their study showed that Celastrol effectively suppressed food intake and increased leptin sensitivity, resulting in weight loss of up to 45% in mice with diet-induced hyperleptinemia (DIO). Notably, Celastrol was ineffective in mouse models lacking leptin or its receptor, suggesting its specificity as a leptin sensitizer and its potential as a pharmacological agent to combat obesity [171].

As for the anti-cancer properties of Celastrol, Lee et al. (2011) investigated its effect on radiation-induced cell death in NCI-H460 lung cancer cells. They found that Celastrol enhanced radiation-induced cell death by increasing the expression of p53, an important tumor suppressor protein. Celastrol achieved this effect by phosphorylating specific serine residues on p53 and inhibiting its proteasomal degradation. In addition, Celastrol acted as a radiosensitizer by inhibiting Hsp90, a protein involved in cancer cell survival [172]. Importantly, Celastrol is structurally distinct from another triterpenoid, 6β-acetonyl-22β-hydroxitingenol (TG), highlighting the importance of its MA functional group in radiosensitization [173].

In another study by Tiedemann et al. (2011), the triterpenoid Pristimerin, a Celastrol methyl ester that also contains an MA group, showed an anti-cancer effect against multiple myeloma tumors. Pristimerin inhibited the activation of NF-kB, a transcription factor involved in the survival of cancer cells, thereby suppressing the transcription of the cyclin D2 promoter. This inhibition resulted in selective lethality of Pristimerin against primary myeloma cells and synergistic cytotoxicity with bortezomib, a standard treatment for multiple myeloma [174].

Wang et al. (2015) discovered the inhibition of the c-Myc oncoprotein by Celastrol, which plays a key role in many human cancers. Celastrol disrupted the quaternary structure of the preformed c-Myc-Max dimer, preventing it from binding to DNA and inhibiting cancer cell proliferation. Remarkably, Celastrol and its derivatives showed high specificity and efficacy against c-Myc, even in neuroblastoma cells overexpressing n-Myc, another member of the Myc family [175]. These results underline the potential of Celastrol as a valuable tool for the development of new cancer therapies.

**Figure 21:**
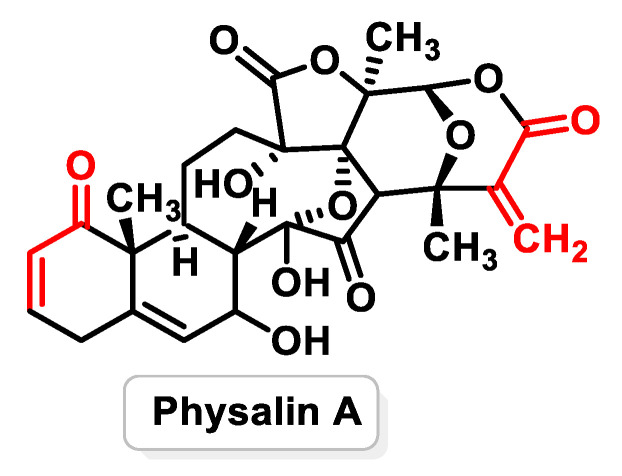
Physalin A molecular structure.

Withanolides, on the other hand, are a class of naturally occurring steroids found mainly in the nightshade family. Structurally, they consist of a steroid scaffold bound to a lactone or its derivatives. Withanolides are traditionally used in Ayurvedic medicine and show promise for various health applications such as inflammation, stress, immunity and vitality. Physalin A (Figure 21) and Physalin B, isolated from Physalis species, are among the known Withanolides with potential therapeutic benefits [176].

Physalin A, a natural compound found in Physalis species, has shown strong anti-cancer properties through various mechanisms. A study conducted by Fanfan Zhu et al. (2016) [177] showed that Physalin A exhibited anti-tumor activity in non-small cell lung cancer (NSCLC) cell lines by targeting the JAK/STAT3 pathway. Physalin A effectively suppressed constitutive and induced STAT3 activity by inhibiting the phosphorylation of JAK2 and JAK3. In addition, Physalin A prevented the nuclear translocation and transcriptional activity of STAT3, resulting in a lower expression of STAT3 and its downstream target genes such as Bcl-2 and XIAP [178].

Physalin A also possesses anti-inflammatory properties by targeting specific residues in IKK-β, a protein involved in the NF-κB signaling pathway. By modulating IKK-β, Physalin A can inhibit NF-κB activation and thereby reduce inflammation [179]. In addition, Physalin F, another Physalin A-related compound, induces apoptosis in human renal cancer cells (A498) via the ROS-mediated mitochondrial pathway and suppresses NF-κB activation [180].

**Figure 22:**
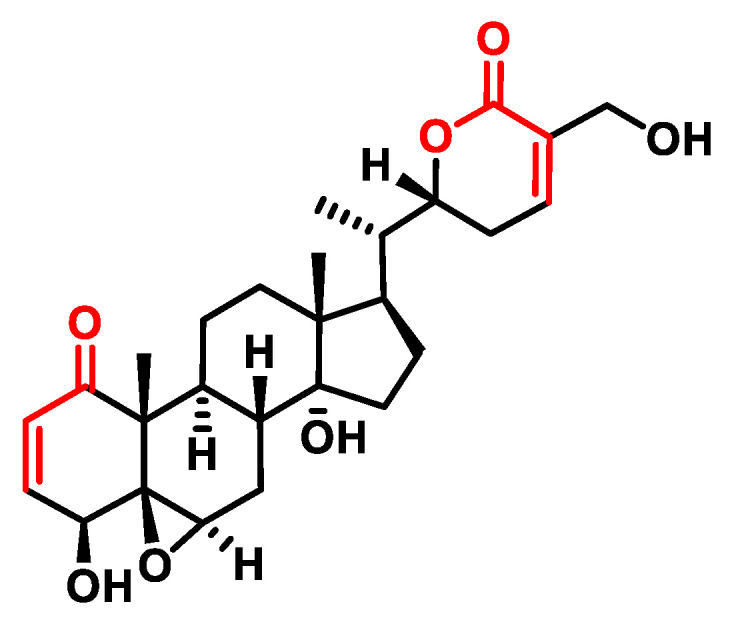
Withaferin A molecular structure.

Withaferin A, (Figure 22), which is extracted from plants such as ashwagandha, belongs to the Ergostane class of Withanolides. Withaferin A has been investigated for its various pharmacological activities, including its anti-cancer properties. However, the specific mechanisms by which Withaferin A exerts its effects are not yet fully understood.

Withaferin A, a natural compound found in plants such as ashwagandha, has shown promise as a therapeutic agent due to its diverse pharmacological activities. One of its remarkable properties is its anti-cancer activity, which has been extensively studied both in vitro and in vivo. Withaferin A has been found to interact with key molecules involved in carcinogenesis, leading to its pro-apoptotic effect [181].

Research by Royce Mohan et al. (2004) has shown that Withaferin A inhibits angiogenesis, the process of new blood vessel formation that is critical for tumor growth. By inhibiting the sprouting and proliferation of human umbilical vein endothelial cells (HUVECs), Withaferin A impairs the formation of new blood vessels and thus suppresses tumor growth and metastasis [182].

In addition, Withaferin A has been shown to inhibit activation of the NF-κB signaling pathway, leading to cell cycle arrest in the G2/M phase and subsequent apoptosis or cell death. This mechanism is thought to be responsible for its anti-cancer effect in various types of cancer [183].

In hepatocellular carcinoma (HCC) cells, Withaferin A was investigated as a regulator of the liver X-α receptor (LXR-α), a nuclear receptor involved in the regulation of gene expression. Varsha D. Shiragannavar et al. (2021) showed that Withaferin A inhibits the proliferation, migration, invasion and anchorage-independent growth of HCC cells. In addition, it downregulates NF-κB, angiogenesis and secretion of inflammation-associated proteins associated with HCC development and progression [183].

In addition, Withaferin A has shown potential as an inhibitor of the key SARS-CoV2 protease Mpro, suggesting a potential role in combating COVID-19 [184].

Toxicity studies have shown Withaferin A to be well tolerated in animals with no evidence of hepatotoxicity, mutagenicity or carcinogenicity. This safety profile supports its potential therapeutic use [185].

**Figure 23:**
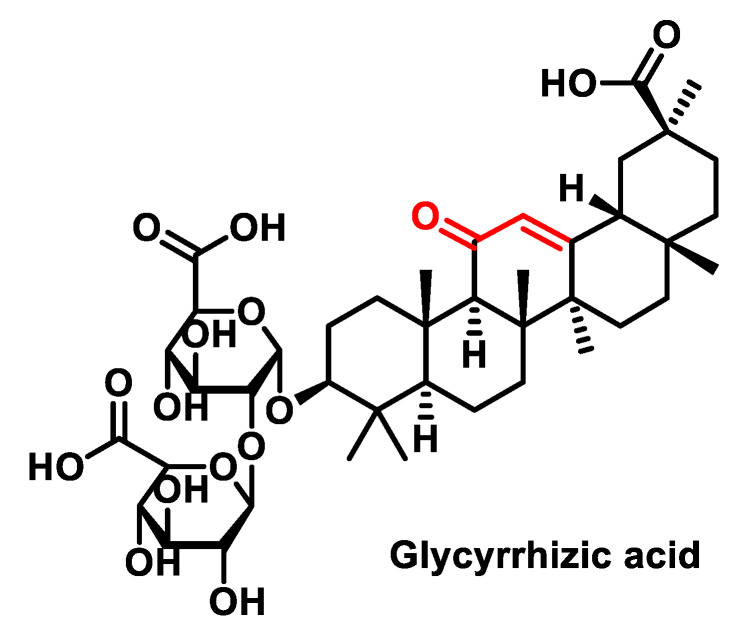
Glycyrrhizinic acid molecular structure.

Saponins, such as Glycyrrhizic acid from licorice, (Figure 23), have also shown anti-cancer properties by inducing the mitochondrial apoptotic pathway and the production of reactive oxygen species (ROS) in breast cancer cells. This underlines the potential of natural compounds such as saponins in cancer therapy [185].

#### 5.2.4. Tetraterpenoids

β-Carotene and fucoxanthin are two remarkable carotenoids with diverse biological activities and potential therapeutic applications.

β-carotene, a deep orange-red pigment found in plants and fruits, is characterized by having beta rings at both ends of the molecule. It is synthesized from geranyl pyranophosphate and belongs to the carotenes, which are terpenoids consisting of eight isoprene units. β-carotene is known for its antioxidant properties, which contribute to its potential health benefits [186].

**Figure 24:**
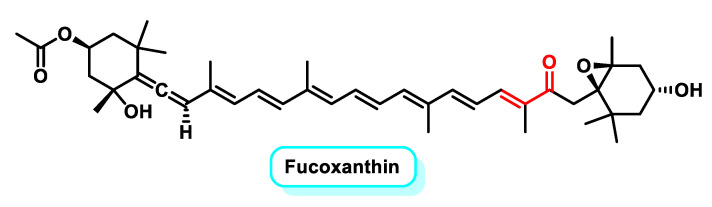
Fucoxanthin molecular structure.

Fucoxanthin (Figure 24), on the other hand, is a brownish carotenoid pigment found in algae such as *Phycophytes* spp. and *Chrysophyta phaeophyceae*.

It has unique chemical structures, including an allenic bond, epoxide and hydroxyl groups, which contribute to its antioxidant activity [187]. Studies have shown that supplementation with fucoxanthin can increase the activity of antioxidant enzymes and reduce liver and plasma triglyceride concentrations, cholesterol levels and the activities of cholesterol-regulating enzymes [188].

In addition, Fucoxanthin has anti-angiogenic, hepatoprotective, cardiovascular and cerebrovascular protective properties [189,190,191,192,193]. It has been shown to induce G1 cell cycle arrest and apoptosis in cancer cell lines. Fucoxanthinol, a metabolite of fucoxanthin, has been shown to downregulate PPARγ and suppress adipocyte differentiation. Fucoxanthin and Fucoxanthinol induce apoptosis in HTLV-1-infected T cell lines and ATL cells by reducing the expression of various anti-apoptotic proteins [194].

In addition, studies have shown that fucoxanthin inhibits cancer cell proliferation by downregulating the STAT3/EGFR signaling pathway and increasing the expression of cleaved caspase-3, a marker for apoptosis. This suggests that fucoxanthin may have therapeutic potential for cancer treatment [195].

**Figure 25:**
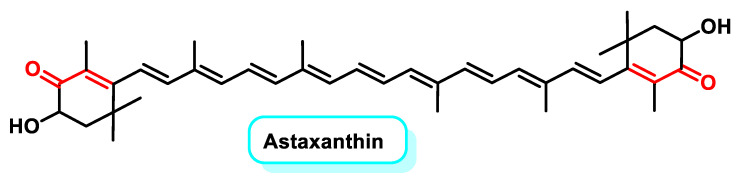
Astaxanthin molecular structure.

Astaxanthin (ASX) is a liposoluble carotenoid belonging to the phytochemical series of terpenes and is classified as a xanthophyll (Figure 25). It is naturally synthesized by numerous microalgae, yeasts and bacteria as secondary metabolites [196].

It has strong antioxidant properties and numerous research studies have shown it to be a more important antioxidant agent than vitamin C, vitamin E and β-carotene [197]. Studies show that ASX reduces the negative effects of aging by neutralizing various reactive oxygen species (ROS) and nitrogen species (RNS) within cells, reducing the oxidative overload of defense systems and the resulting oxidative damage [198].

**Figure 26:**
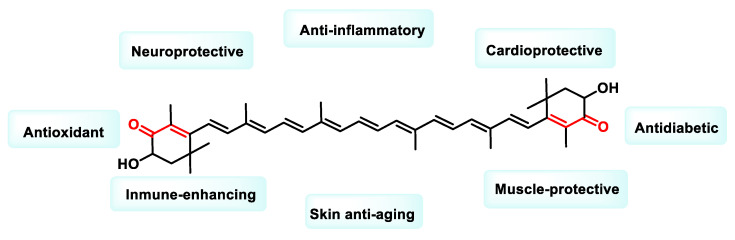
Bioactivity of ASX.

ASX has a several times greater effect than β-carotene in quenching singlet oxygen and the antioxidant function against lipid peroxidation is up to 100 times more significant than in vitamin E. ASX is 6000 times more effective than vitamin C, 770 times more active than coenzyme Q10 (CoQ10), 100 times more potent than vitamin E [199] and five times more potent than β-carotene in scavenging the energy of singlet oxygen, one of the most common ROS in the human body [200,201]. Below is a summary of the biological activities mediated by ASX (Figure 26).

The anti-aging properties of ASX are well known and are attributed to its antioxidant and anti-inflammatory effects, which prevent age-related muscle breakdown and improve mitochondrial energy production [202]. ASX exhibits high safety and bioavailability, making it a potential therapeutic agent for cardiovascular, age-related, neurodegenerative, respiratory and liver diseases [203].

Research by K. Kavitha et al. in 2013 showed that ASX inhibits the NF-κB and Wnt signaling pathways by reducing the activity of the key regulatory enzymes IKKβ and GSK-3β. The Wnt signaling pathway, which is crucial for cellular communication, is closely linked to tumorigenesis due to its abnormal activation observed in many cancers. ASX also promotes apoptosis by modulating the expression of apoptosis-related proteins such as Bcl-2, p-Bad, Bax and Bad [204]. Recent studies by Davinelli et al. (2022) have further investigated the molecular mechanisms underlying the regulation of NRF2 and NF-κB by ASX [205].

In a study conducted by Shao-Qian Sun et al. in 2020, ASX showed anti-cancer effects on prostate cancer (PCa) cells, especially DU145 cells. Treatment with ASX reduced cell proliferation, enhanced apoptosis and inhibited the cells’ ability to migrate and invade. In addition, ASX reduced the expression of STAT3 at both protein and mRNA levels [206].

Further investigation revealed that ASX directly upregulated PPAR-γ, resulting in the inhibition of proliferation, decreased viability, induction of apoptosis and the disruption of cell cycle progression in K562 leukemia cells [207].

Taken together, these results suggest that ASX exerts chemopreventive effects by targeting key molecules in oncogenic signaling pathways and inducing apoptosis, indicating its potential as a candidate for cancer prevention and therapy.

#### 5.2.5. Hop-Derived Bitter Acids

**Figure 27:**
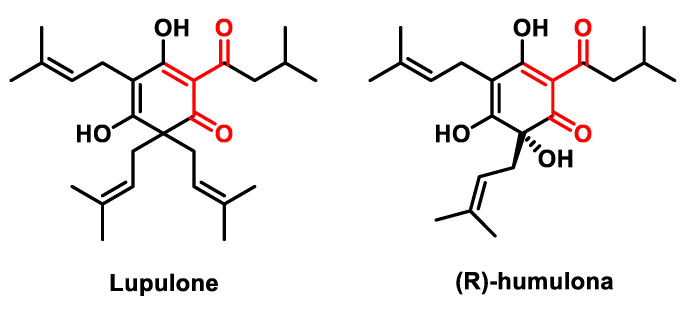
α-acids and β-acids molecular structures.

Hop-derived bitter acids (HBAs) (Figure 27) are a group of organic compounds found in the cones of the hop plant, *Humulus lupulus*, that are responsible for the bitterness in beer and have several potential health benefits.

HBAs are terpenophenolic compounds consisting of isoprene units and phenolic groups. There are two main types: α-acids (Humulones) and β-acids (Lupulones). During brewing, α-acids are converted to iso-α-acids, the main bitter compounds in beer, while β-acids are converted to Hulupones, which contribute to beer flavor and aroma [208].

Apart from their role in brewing, HBAs exhibit antioxidant, anti-inflammatory and antimicrobial properties. Numerous studies, including human studies, have demonstrated their positive effects on weight gain, lipid metabolism, glucose homeostasis, insulin sensitivity and inflammation, suggesting a potential role in the prevention of metabolic syndrome and related diseases [209].

Humulone, a type of HBA, inhibits the catalytic activity and gene transcription of cyclooxygenase-2 (COX-2), which is essential for the regulation of angiogenesis. It also suppresses the proliferation of endothelial cells, the production of vascular endothelial growth factor (VEGF) and the formation of vascular endothelial cells [210]. Studies suggest that humulone may inhibit the promotion of chemically induced skin tumors in mice by blocking the activation of NF-κB and AP-1, which are involved in carcinogenesis, thereby reducing COX-2 expression associated with inflammation and tumor development [211].

Lupulone, another HBA, has been investigated for its potential chemopreventive effect in cancer. Studies in human colon cancer cells and a rat model of colon carcinogenesis revealed that Lupulone significantly inhibited cell growth and upregulated apoptosis-inducing receptors, suggesting an involvement in the apoptosis pathways [212]. In addition, Lupulone increased the permeability of mitochondrial membranes, further supporting its potential anti-cancer effect [213].

### 5.3. Polyketides

Polyketides form a broad class of natural substances with different chemical structures and biological activities. They are synthesized via the polyketide synthase (PKS) pathway, which involves the stepwise condensation of acetyl-CoA units to form a chain of alternating ketone and methylene groups. This basic structure serves as a platform for further modifications, such as the addition of functional groups, cyclization and oxidation, leading to the production of a wide range of polyketides with different properties [214].

Acetogenins, a type of polyketides found in the plants of the *Annonaceae* family, are characterized by linear chains of 32 or 34 carbon atoms containing oxygen-containing functional groups such as hydroxyl groups, ketones, epoxides, tetrahydrofurans and tetrahydropyrans [215]. They are found in various plants such as sorrel, cherimoya, guanabana and breadfruit as well as in some plants from the Vitaceae family such as the grapevine [216]. Acetogenins have a wide range of biological properties:

- They show anti-cancer activity against various types of cancer, including breast, lung, colon and prostate cancer [217].
- They have anti-inflammatory properties that could be useful in the treatment of inflammatory diseases such as rheumatoid arthritis and inflammatory bowel disease [218].
- They have antiviral activity against viruses such as HIV, HCV and HSV [219,220,221,222].
- They have antiparasitic activity against parasites, including the parasite that causes malaria [223].

**Figure 28:**
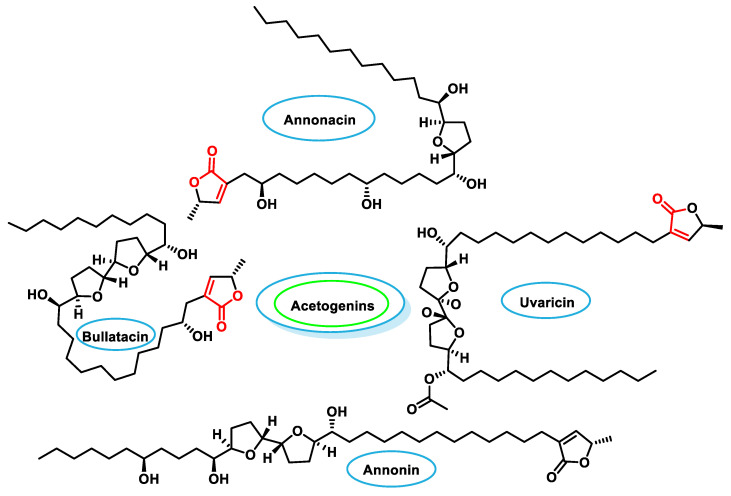
Molecular structures of Annonacin, Annonin, Bullatacin and Uvaricin.

Examples of acetogenins are Annonacin (abundant in sorrel), Annonin (found in sorrel and other *Annonaceae* plants), Bullatacin (found in cinnamon apple) and Uvaricin (found in breadfruit). The *Annonaceae* acetogenins inhibit complex I in the respiratory chain of tumor cells, leading to decreased ATP production, while modulating the expression of apoptosis-related proteins such as caspases and Bcl-2 to induce apoptosis in cancer cells [224] (Figure 28).

Studies have shown that treatment with acetogenins promotes apoptosis and arrests the cell cycle in various cancer cell lines while downregulating multidrug resistance proteins. In addition, acetogenins lower levels of NF-κB and Akt, which are involved in cancer cell proliferation and survival [225].

**Figure 29:**
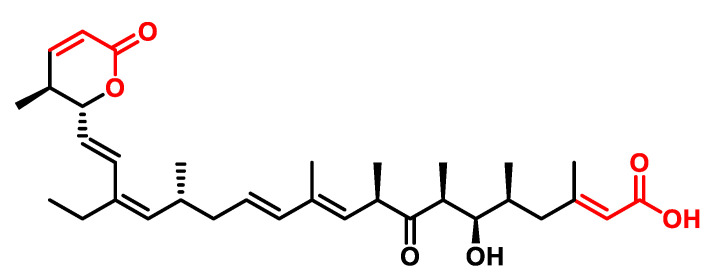
Leptomycin B molecular structure.

Leptomycins, secondary metabolites produced by *Streptomyces* spp., in particular leptomycin B (LMB) (Figure 29), were initially identified as potent antifungal compounds [226]. However, recent research has revealed their potential as antitumor agents. LMB induces G1 cell cycle arrest and inhibits nuclear export by binding to XPO1, the primary receptor for the nuclear export of proteins and RNA [227]. It also inhibits the export of various proteins and RNAs, including HIV-1 Rev, MAPK/ERK, NF-κB, COX-2 and c-Fos mRNAs, by covalently binding to Cys-529 of XPO1 [228].

In *Schizosaccharomyces pombe*, Leptomycin B (LMB) is known for its ability to interfere with nuclear export by interacting with XPO1, the primary receptor responsible for the transport of proteins and RNA from the nucleus. LMB binds to the cysteine residue Cys529, which is in the NES binding cleft of XPO1 and thus impedes its function. In 1999, Nobuaki Kudo et al. demonstrated that LMB forms a covalent bond via a Michael-type addition with N-acetyl-L-cysteine methyl ester, suggesting that LMB binds to the sulfhydryl group of Cys-529 via its α,β-unsaturated δ-lactone [228].

In addition, at low nanomolar concentrations, LMB inhibits the nuclear export of numerous proteins, including HIV-1 Rev [229], MAPK/ERK [230] and NF-κB [231], while inhibiting the inactivation of p53 [232]. LMB also inhibits the export and translation of various RNAs, such as COX-2 and c-Fos mRNAs, by interfering with the export of ribonucleoproteins [233].

### 5.4. Polyphenols

Polyphenols are a class of compounds that occur frequently in plants and are characterized by the presence of several phenolic groups in their chemical structure. These compounds have aromatic rings to which hydroxyl groups (-OH) are attached. They are abundant in various plant foods, including fruits, vegetables, nuts, seeds, whole grains and legumes. Foods rich in polyphenols include a wide range of plant sources. Polyphenols have various health-promoting properties, including antioxidant, anti-inflammatory, cardioprotective, neuroprotective and anti-carcinogenic effects [234]. The recommended daily intake of polyphenols is generally between 250 and 500 mg. This amount can be achieved through a diet rich in fruits, vegetables, nuts, seeds, whole grains and legumes.

Numerous polyphenolic compounds have MA groups, which we will discuss in more detail in the following sections.

#### 5.4.1. Esters Derived from Caffeic Acid

Caffeic acid, known for its potential health benefits, is an organic compound that belongs to the group of hydroxycinnamic acids. This compound, which appears as a yellow solid, contains both phenolic and acrylic functional groups. It is ubiquitous in plants as it serves as an intermediate in the synthesis of lignin, a major component of woody plant biomass and its residues [235]. Caffeic acid can be extracted from various plant species, including the bark of *Eucalyptus globulus*, barley grains (*Hordeum vulgare*), the herb *Dipsacus asperoides*, the freshwater fern *Salvinia molesta* and the fungus *Phellinus linteus*. It is also found in numerous beverages such as coffee and red wine as well as in foods such as fruit, spices, sunflower seeds, apple sauce, apricots, plums and black chokeberries [236]. Caffeic acid can form several natural esters, including the following:

- Caffeic acid phenethyl ester (CAPE), a phenolic compound recognized as one of the major active components in propolis, with significant biological activities [237].
- Dactylifric acid (DA), also known as date acid or 5-O-caffeoylshikimic acid, an ester derived from caffeic acid and shikimic acid, found mainly in dates (*Phoenix dactylifera* fruits) [238].
- Chlorogenic acid (CGA), the ester formed from caffeic acid and quinic acid. The collective term “chlorogenic acids” refers to a family of related polyphenols that include hydroxycinnamic acids bound to quinic acid (such as caffeic acid, ferulic acid and p-coumaric acid). Chlorogenic acids are widely used in foods such as coffee beans, coffee drinks, mate and tea and include numerous isomers, each with different sensory properties [239].
- Rosmarinic acid (RA), isolated from *Rosmarinus officinalis* L., and commonly known as rosemary.

**Figure 30:**
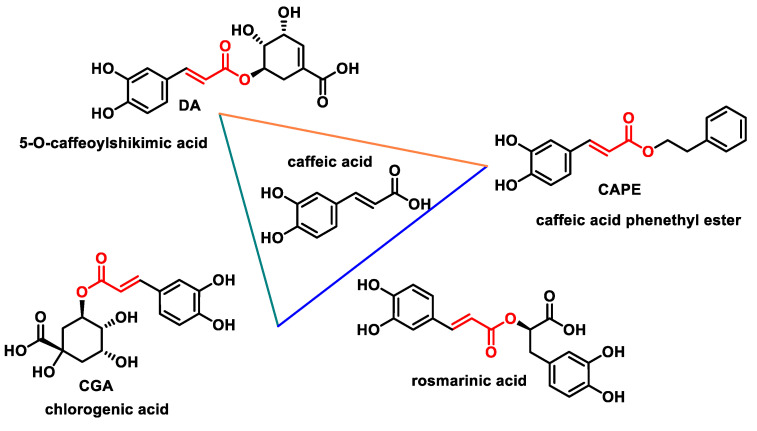
Molecular structure of CAPE, DA, CGA and RA.

The molecular structure of the four esters is presented below (Figure 30).

CAPE, a major bioactive component of propolis, shows potential efficacy in the treatment of various cancers, including breast, colon, lung and prostate cancer [240]. In relation to breast cancer, studies have shown that CAPE interferes with the metastasis of breast cancer cells by deactivating FGFR1 via MD2. These studies suggest that CAPE impedes the migration, invasion and epithelial–mesenchymal transition (EMT) of breast cancer cells. Mechanistically, CAPE inhibits FGFR1 phosphorylation and nuclear translocation, while overexpression of FGFR1 attenuates the anti-metastatic effects of CAPE [241]. Another study has shown that combination therapy of docetaxel with CAPE suppresses the survival and proliferation of docetaxel-resistant prostate cancer cells through apoptosis induction and metabolic interference [242].

CAPE also attenuates the pro-inflammatory and fibrogenic phenotypes of lipopolysaccharide (LPS)-stimulated hepatic stellate cells by inhibiting NF-κB signaling [243]. In vivo experiments show that CAPE administered intraperitoneally at a dose of 10 mg/kg inhibits the growth and angiogenesis of primary tumors in mice inoculated with Lewis lung carcinoma, colon carcinoma and melanoma cells [244].

These results highlight the potential of CAPE as a drug candidate for cancer treatment due to its robust anti-tumor effect with minimal toxicity.

DA, also known as 5-O-caffeoylshikimic acid, is a plant-derived bioactive compound being investigated for its potential in cancer treatment, particularly in non-small cell lung cancer (NSCLC). It exhibits increased binding affinity to the MEK protein (mitogen-activated protein kinase, or MAPK), a critical component of the MAPK signaling pathway that regulates gene expression, cell growth and survival [245]. Dysregulated MAPK signaling often leads to uncontrolled cell proliferation and resistance to apoptosis, contributing to cancer development. MEK inhibition is emerging as a promising strategy for the treatment of NSCLC due to the diverse structures and deleterious effects of upstream receptors in the MAPK signaling pathway [246].

CGA was found to attenuate the malignant properties of hepatocellular carcinoma (HCC) cells by suppressing DNMT1 expression, which is associated with the inactivation of tumor suppressor genes. CGA inhibits the proliferation, colony formation, invasion and metastasis of HepG2 cells (a type of HCC cells) both in vitro and in vivo [247]. In addition, CGA induces apoptosis and cell cycle arrest in colorectal cancer cells and reduces cell viability in a dose-dependent manner. This mechanism involves cell cycle arrest by upregulating the expression of p21 and p53, inducing apoptosis by downregulating the expression of Bcl-2 and NF-κB and increasing the expression of caspase 3 and 9 and ROS levels [248].

RA inhibits the growth of human cancer cell lines, including small cell lung carcinomas, as well as human breast and prostate adenocarcinomas [249].

#### 5.4.2. Chalcones

**Figure 31:**
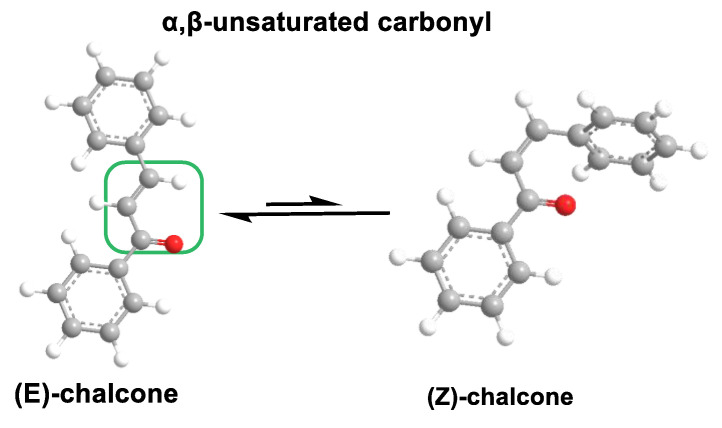
Trans and cis isomers of the basic chalcone skeleton.

Chalcones are open-chain flavonoids characterized by three carbons between two aromatic rings linked by an α,β-unsaturated carbonyl system. These compounds, aromatic ketones, serve as the basic structure for numerous vital biological compounds and are the biogenetic precursors of flavonoids and isoflavonoids, which are abundant in plants. Chalcones occur as both trans and cis isomers, with the trans isomer (E) being the most thermodynamically stable configuration (Figure 31).

Chalcones are widely used in vegetables, fruits, teas and various other plants and are isolated from species belonging to the *Leguminosae*, *Asteraceae* and *Moraceae* families. Due to their known bioactivity and low toxicity profile, they have been extensively studied over the years [250,251,252].

**Figure 32:**
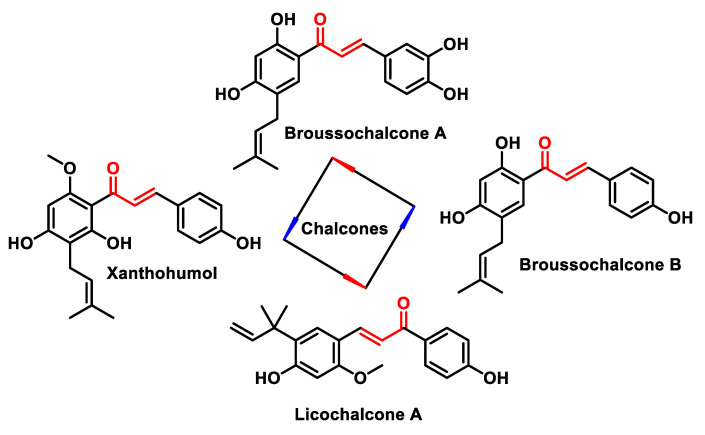
Molecular structure of Xanthohumol, Brussochalcone A, Brussochalcone B and Licochalcone A.

The biological effects of chalcones, (Figure 32), are primarily due to the presence of an α,β-unsaturated carbonyl group, which endows them with the properties of MA compounds [251,253,254]. This reactive functional group readily forms covalent bonds with nucleophiles, such as the sulfhydryl groups of cysteine residues in peptides or cell proteins, leading to the formation of Michael adducts.

Chalcones have promising potential in cancer therapy, as they show efficacy against various types of cancer both in vitro and in vivo through different mechanisms. These mechanisms include interrupting the cell cycle, regulating autophagy, inducing apoptosis, modulating immunomodulatory responses and influencing inflammatory mediators, all with low toxicity [255].

Heat shock protein 90 kDa (HSP90) is a chaperone protein that is critical for proper protein folding, stabilization against hyperthermia and protein degradation. It plays an important role in the acquired capabilities of cancer cells. Recent studies have shown that synthetic chalcones can inhibit HSP90 activity [256,257]. Phase II clinical studies using chalcones with hydroxyl groups at the 1,3 positions have shown inhibition of HSP90 interactions with patient proteins by binding to the ATP site of HSP90 [258].

In the field of medicinal chemistry, chalcone–metal complexes have attracted attention due to their ability to chelate various metals and their regulatory effects on multiple cancer targets [259]. Research has shown that novel Ru(II)–chalcone complexes have considerable activity against breast cancer through inhibition of DNA topoisomerase [260].

Numerous studies have reported that chalcones induce cell cycle arrest in the G2/M phase. For example, certain chalcone–acridine hybrids and quinoline–chalcone hybrids have shown efficacy in inhibiting melanoma cell progression by inducing G2/M cell cycle arrest, DNA damage, apoptosis and the modulation of MAP kinase activity [261]. In addition, treatment with 2′,4′-dihydroxychalcone has led to an accumulation of G2/M phase in human gastric carcinoma cells [262].

Licochalcone A (LCA), found in the root of *Glycyrrhiza glabra* L. or *Glycyrrhiza infate*, has shown the ability to inhibit the growth of oral squamous cell carcinoma cells in a dose-dependent manner and inhibits specificity protein 1 (Sp1). Similarly, polyphenols isolated from *Brussonetia papyrifera*, including Broussochalcone B and Broussochalcone A, have shown anti-cancer potential via the activation of FOXO3 [263].

Xanthohumol (XH), a prenylated chalcone from *Humulus lupulus*, inhibits COX-1 and COX-2 activity and has chemopreventive properties [264]. It also acts as a powerful antiviral agent against various viruses and induces growth inhibition and apoptosis in cancer cells. XH has been shown to inhibit invasion and cell cycle progression in cancer cells, specifically targeting the MMP2, MMP9, FAK and p53 genes in three-dimensional breast and lung cancer cell cultures [265].

#### 5.4.3. Curcuminoids

Curcuminoids, found in turmeric, a spice native to India and Southeast Asia, have attracted considerable attention for their potential health benefits. They have been extensively studied for their anti-inflammatory and antioxidant properties and have been an integral part of traditional Indian medicine for centuries, prized for their medicinal properties [266].

**Figure 33:**
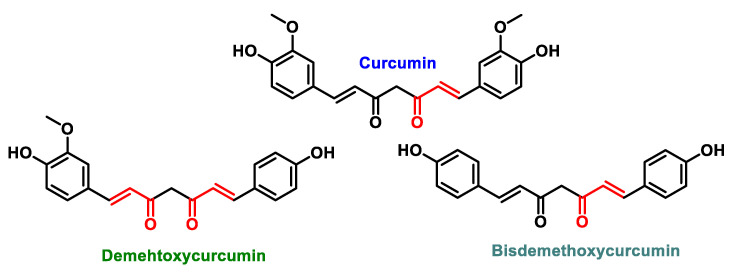
Molecular structure of Curcumin, Demethoxycurcumin and Bisdemethoxycurcumin.

Curcuminoids, known for their potent antioxidant and anti-inflammatory effects, have also been studied for their potential neuroprotective, anti-tumor, anti-acidogenic, radioprotective and anti-arthritic properties [267]. Their diverse spectrum of biological activities underscores their importance for various health-related applications. The most important curcuminoids are shown in Figure 33.

Numerous studies suggest that curcuminoids may offer therapeutic benefits in various chronic diseases such as colorectal cancer, lung cancer, breast cancer and inflammatory bowel disease [267]. Curcuminoids have a wide range of potential health applications, including the following:

- Protecting cells from free radical damage that contributes to aging and disease [268].
- They show neuroprotective effects by protecting brain cells from oxidative stress and inflammation, both of which are associated with neurodegenerative diseases such as Alzheimer’s and Parkinson’s [269].
- They have anti-inflammatory properties that are crucial in combating chronic inflammation such as arthritis, cancer and cardiovascular disease [270].
- They have shown anti-tumor properties in laboratory studies, which has led to ongoing clinical trials to investigate their effectiveness in cancer treatment [271].
- They have an anti-acidifying effect, which may be beneficial for people with heartburn or acid reflux as it reduces gastric acid secretion.
- They act as a radioprotective agent by protecting cells from radiation-induced damage, which is particularly beneficial for people undergoing radiotherapy for cancer [272].
- They relieve arthritis symptoms by reducing inflammation and associated pain [273].

**Figure 34:**
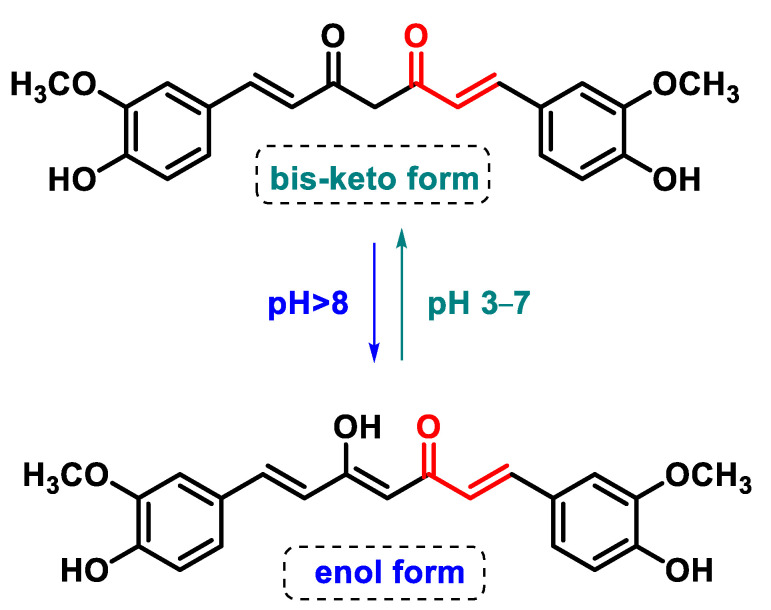
Tautomerism of curcumin.

The most well-known curcuminoid is curcumin (Figure 34) with a distinctive yellow color.

Curcumin serves as a natural activator of the NRF2 signaling pathway due to its electrophilic α,β-unsaturated carbonyl residues that can covalently bind to a cysteine residue of Keap1 [11]. It acts as an inhibitor of p300 histone acetyltransferase (with an IC50 of about 25 μM) [274] and histone deacetylase (HDAC) [275] while suppressing NF-κB activation [276]. In addition, curcumin induces mitophagy, autophagy, apoptosis and cell cycle arrest, thus exhibiting antitumor activity. In renal damage associated with rhabdomyolysis, curcumin reduces ferroptosis-mediated cell death [277,278]. In addition, curcumin exhibits anti-infective properties against various human pathogens, including the influenza virus, hepatitis C virus, HIV and strains of *Staphylococci*, *Streptococci* and *Pseudomonas* [279].

Its suppression of NF-κB activity occurs by inhibiting the phosphorylation of IκB and blocking the nuclear translocation of the NF-κB p65 subunit [280]. Curcumin exerts its anti-cancer effects observed in various in vitro models through the inhibition of AP-1 and NF-κB factors [281]. STAT3 emerges as a molecular target of curcumin in numerous tumors, both directly and indirectly via the inhibition of IL-6 [282,283]. Curcumin also regulates cyclin D1 levels, an important regulator of cell cycle progression with implications for cancer development and progression. Its ability to suppress cyclin D1 is mediated through the inhibition of NF-κB [284].

#### 5.4.4. Flavonoids

Flavonoids, derived from the Latin word flavus meaning “yellow”,” represent a group of secondary plant metabolites that are synthesized from a phenylalanine molecule and three malonyl-CoA molecules via the flavonoid biosynthetic pathway. The resulting basic structure, characterized by a cyclic C6-C3-C6 skeleton, serves as the basis for a diverse family of compounds known for their polyphenolic character [285].

**Figure 35:**
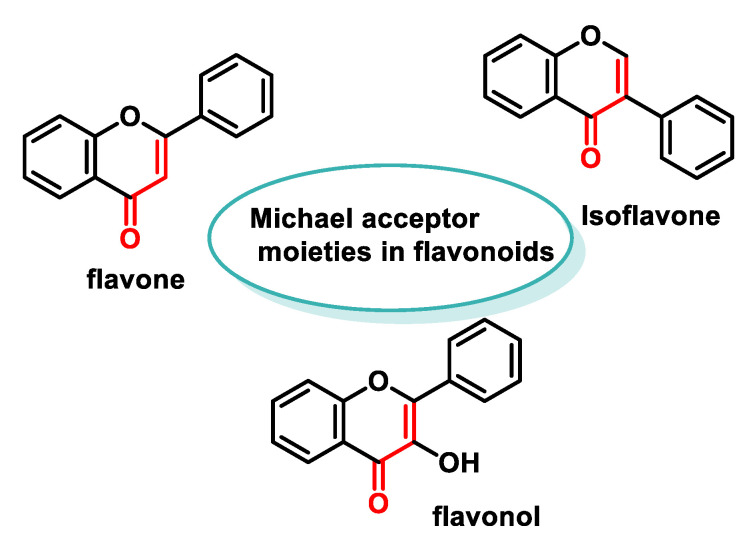
Michael acceptor moieties in flavonoids.

Certain flavonoids (Figure 35) exhibit anti-cancer properties against various human cancer cell lines, including cervical (HeLa), hepatoma (Hep-G2) and breast cancer (MCF-7) cell lines [286].

Flavonols, such as myricetin and Kaempferol, are subclasses of flavonoids found in a variety of fruits and vegetables. These compounds have attracted attention due to their potential anti-cancer effects mediated by the modulation of various tumorigenic mechanisms [287]. For example, astragalin, a Kaempferol derivative, inhibits the proliferation and migration of human colon cancer cells via the NF-κB signaling pathway, induces cancer cell apoptosis and arrests the cell cycle [288]. Kaempferol itself plays a chemopreventive role by inhibiting carcinogenesis and cancer progression while suppressing metastasis-related behaviors in breast cancer cells [289].

Isoflavones, another subclass of flavonoids found in soy, have been investigated for their potential anti-cancer effects [290]. Studies suggest that higher soy consumption, which is mainly due to soy isoflavones, is inversely related to the incidence of cancer. Alpinum isoflavone, an isoflavone extracted from *Erythrina suberosa Roxb*., shows cytotoxic effects against human leukemia cell lines [291].

Flavones, another important subclass of flavonoids, are abundant in various plants and have shown anti-cancer activity against human breast, liver and lung cancer cells [292]. Apigenin, a well-researched flavone, exhibits a broad spectrum of anti-cancer effects in various cancers, including colon, breast, liver, lung, melanoma, prostate and osteosarcoma cancers. Its mechanisms include the inhibition of neoplastic cell proliferation, the induction of cell apoptosis and autophagy, the modulation of the cell cycle and the suppression of cancer cell motility, migration and invasion [293,294,295,296,297,298].

**Figure 36:**
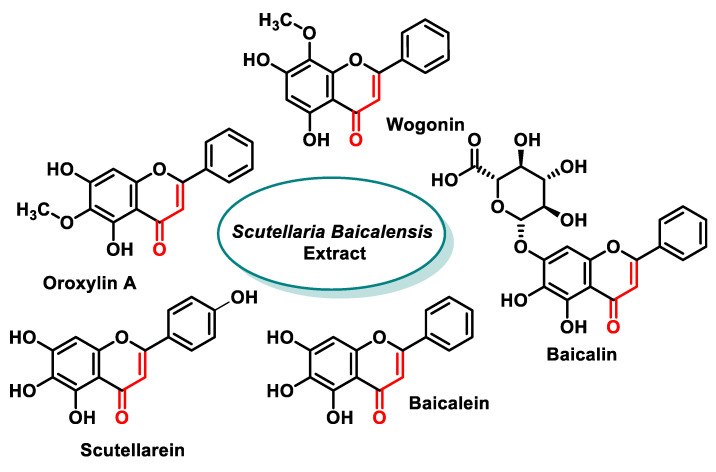
Flavones in *Scutellaria baicalensis* extract.

*Scutellaria baicalensis* (SB) extract contains several flavones that can inhibit cell invasion and regulate the cancer cell cycle [299]. The main components of this extract are listed in Figure 36.

Wogonin, extracted from the roots of *Scutellaria baicalensis* [300], serves as the main bioactive compound with a variety of pharmacological activities, including antiviral [301], antioxidant, antibacterial, anti-cancer [302], anxiolytic [303] and neuroprotective [304] effects. Specifically in ovarian cancer, wogonin primarily inhibits cell growth and promotes apoptosis [305].

Baicalein induces apoptosis in A2780 cells via the intrinsic apoptotic pathway by enhancing the activity of caspase-3 and PARP. In combination with paclitaxel, baicalein further promotes apoptosis in human ovarian cancer cells [306].

Baicalin, a glycosidic flavonoid found in various plant families such as *Lignanaceae* and *Labiatae* and mainly isolated from *Scutellaria* species, has antiproliferative potential and modulates various signaling pathways [307].

Oroxylin A, a flavonoid extracted from *Scutellaria baicalensis*, *Oroxylum indicum* and other plants, exerts its effects by modulating various signaling pathways, including PTEN/PI3K/Akt, NF-κB, MAPK and Wnt/β-catenin [308].

Scutellarein shows anti-cancer effects in various human cancers [309]. In OC cell lines A2780 and SKOV-3, treatment with Scutellarein reduces proliferation rates, especially at a concentration of 100 μM after 48 hours, while inhibiting cancer cell migration and invasion [310].

#### 5.4.5. Coumarins

**Figure 37:**
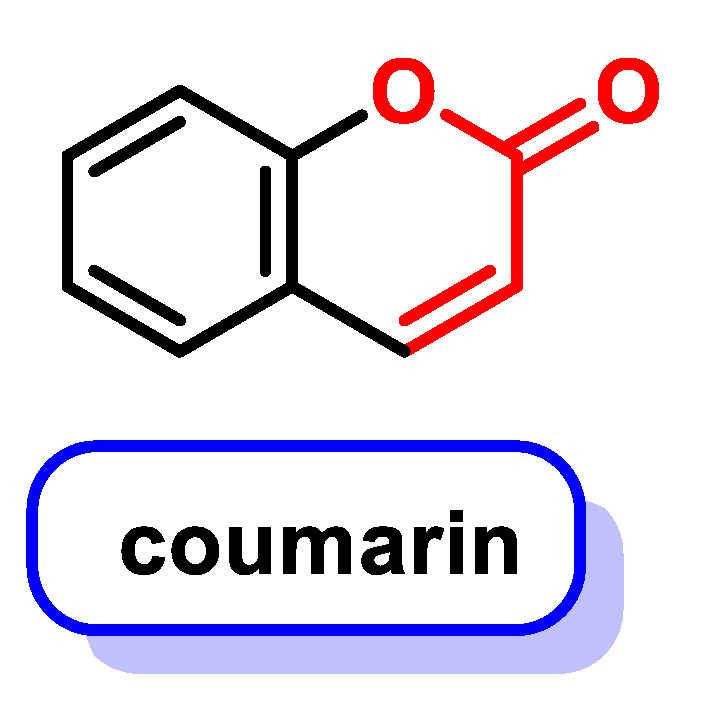
Coumarin structure.

Coumarins, which originate from the shikimic acid pathway, are phenolic compounds that are lactones of either natural or synthetic origin. These derivatives form a family of heterocyclic compounds characterized by the presence of a 1-benzopyran-2-one backbone, also known as 2H-chromen-2-one, which harbors an electron-rich, conjugated π-π-π system [311]. Coumarin (Figure 37), an aromatic organic chemical compound with the molecular formula C9H6O2, is widely distributed in many plants, where it serves as a chemical defense mechanism against predators [312]. A notable derivative is the prescription drug warfarin, which inhibits the formation of blood clots, thereby attenuating conditions such as deep vein thrombosis and pulmonary embolism by inhibiting vitamin K synthesis [313].

Natural coumarin and its derivatives are widely distributed and occur in various sources, including certain fungi such as *Basidiomycetes* and *Ascomycetes* as well as numerous plant species from different botanical families such as *Fabaceae*, *Rubiaceae*, *Rutaceae*, *Asteraceae*, *Umbeliferae*, *Apocynaceae*, *Compositae*, *Orchidaceae* and *Labiatae* [314]. Coumarins are one of the most abundant natural products and are remarkably diverse.

**Figure 38:**
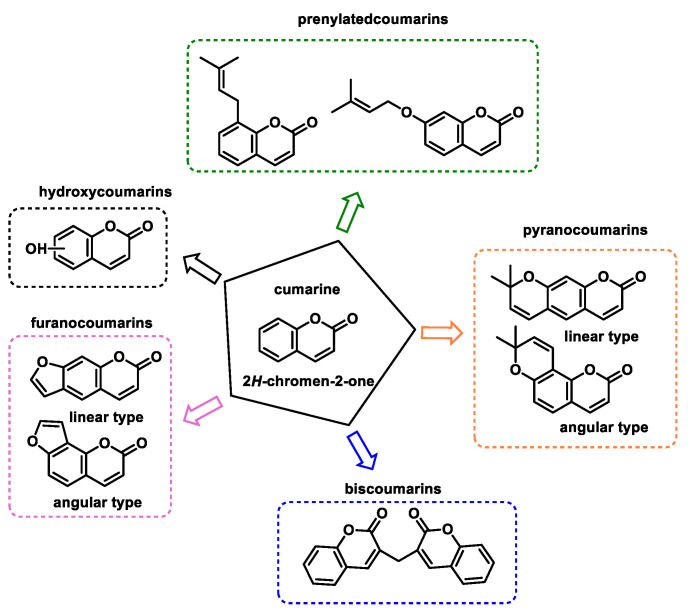
Classification of coumarins according to their chemical structure.

Based on various modifications and substitutions, coumarins (Figure 38) can be divided into simple and complex forms. Coumarins fulfill various physiological functions and contribute to the defense mechanisms against pathogens and insects as well as protection against abiotic stress. They are also recognized as bioactive compounds with therapeutic potential in diseases such as AIDS and various types of cancer.

In addition to the coumarins that have been isolated in plant species and other microorganisms, numerous derivatives of synthetic origin have been developed that considerably expand the repertoire of known coumarin structures and their complexity [315].

The coumarin ring is an essential component of various medical applications and contributes to the development of anti-cancer, antioxidant, antifungal, anticoagulant, anti-inflammatory, antiviral, antibacterial, antiprotozoal and vasodilator compounds. It also serves as an inhibitor of enzymes such as cholinesterase, carbonic anhydrase, monoamine oxidase, serine protease, cyclooxygenase and lipoxygenase [316,317,318,319,320,321,322,323,324,325,326,327,328].

**Figure 39:**
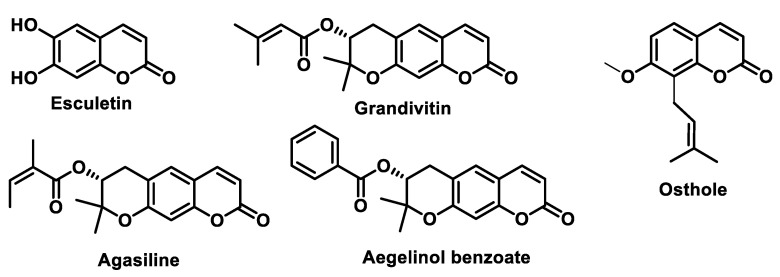
Chemical structures of Esculetin, Grandivitine, Agasiline and Aegelinol benzoate.

These compounds have been studied in cancer cell lines and have shown effects ranging from the inhibition of cell proliferation to interference with various stages of the cell cycle and apoptotic processes. Certain coumarin derivatives, such as Imperatorin and Osthole, show anti-cancer effects by inhibiting the migration and invasion of neoplastic cells. Esculetin has shown antineoplastic activity by protecting cultured primary neurons from the toxicity of N-methyl-D-aspartate. In addition, derivatives such as Grandivitin, Agasilin, Aegelinol benzoate and Osthole have shown cytotoxic effects against the lung cancer cell line A549. The anti-cancer properties of Osthole include inhibition of the proliferation, motility and metastasis of cancer cells as well as the induction of apoptosis and cell cycle arrest in mouse models [329] (Figure 39).

MAs and natural compounds in general have been widely investigated for their anticarcinogenic potential due to their remarkable chemical diversity [330]. However, there are several limitations to their use in the prevention and treatment of cancer:

- Difficulty in large-scale isolation: they are often difficult to isolate in large quantities, which can hinder their development into drugs.
- Understanding the action mechanism can be challenging, which can slow down their pharmaceutical development.
- Pharmaceutical development challenges: even when they show promising anti-cancer properties, it can be difficult to develop them into a drug that can be used in clinical settings.
- Bioavailability and solubility issues of MA, such as curcumin or resveratrol, have shown potent anti-cancer activity but have poor solubility and bioavailability when administered alone.

Despite these challenges, advancements in technology and our growing knowledge of cancer therapy may help overcome these hurdles and improve the efficiency of drug discovery. For example, the use of nanoparticles can enhance the solubility and bioavailability of natural product-derived compounds. Furthermore, understanding the role of non-coding RNAs in the regulation of natural products can potentially broaden their application in cancer therapy [331].

## 6. Antibody and Peptide-Mediated Delivery of Michael Acceptors

The use of Michael acceptors as anti-cancer drugs represents a promising avenue in the ongoing search for effective cancer therapies [332]. These electrophilic compounds exhibit potent cytotoxicity due to their ability to covalently alter critical cellular macromolecules such as proteins and nucleic acids, thereby inducing cell death [333]. However, the clinical translation of Michael acceptors faces challenges related to their delivery, including poor solubility, off-target effects and limited tumor selectivity [334,335]. To address these issues, researchers have increasingly turned to peptide conjugation strategies to target Michael acceptors to cancer cells [336,337,338]. Antibodies and peptides offer several advantages, including high affinity and selectivity for cell surface receptors overexpressed on cancer cells, as well as the ability to facilitate intracellular delivery through receptor-mediated endocytosis [339]. By conjugating Michael acceptors with antibodies and peptides, researchers seek to improve the therapeutic index of these compounds and maximize their efficacy against cancer cells while minimizing systemic toxicity [340]. A recent study investigates the use of monomethyl auristatin E (MMAE) as a potent cytotoxic agent in antibody–drug conjugates (ADCs) for cancer treatment and its ability to sensitize cells to radiation-induced DNA damage. The researchers investigated the efficacy of MMAE in combination with fractionated radiotherapy by conjugating it with two different tumor-targeted drug carriers: antibodies and activatable cell-penetrating peptides (ACPPs). Using the linker Maleimidocaproyl-valine-citrulline-P-aminobenzyl-carbamyl (MC-VC-PABC), MMAE was bound to both carriers so that it could be tested in resistant xenografts, syngeneic tumors and an autochthonous tumor model [341]. Several studies have investigated alternative auristatin analogs as cytotoxic payloads in antibody–drug conjugates (ADCs) for cancer therapy. These include auristatins F, M and W, as well as monomethyl auristatin F (MMAF), PF-06380101, Duostatin 5 and keto-auristatin PE (KAPE). For example, MMAF was used in Belantamab mafodotin ADC for the treatment of relapsed or refractory myeloma [342]. Another Mafodotin-based ADC, Depatuxizumab mafodotin, targets EGFR in glioblastoma and EGFR-overexpressed tumors [343]. In addition, trastuzumab-based ADCs using Dolastatin 10 or related analogs as payloads have shown promising results in targeted therapies for HER2-positive cancers [344]. These studies underscore the diverse applications and potential of auristatin analogs in ADC development for targeted cancer therapy.

## 7. Conclusions

In recent decades, the pharmaceutical industry has shifted away from the development of inhibitors that bind covalently to enzymes or other target proteins via the Michael reaction. This shift was triggered primarily by safety concerns regarding the indiscriminate and non-selective reactivity of covalently modifying drugs with potentially non-target proteins, leading to unpredictable toxicity. However, with the successful approval of several effective and safe covalent protein kinase-binding inhibitors for cancer treatment, interest in this class of drugs has increased again. This has led to a change in perspective regarding the potential of covalent inhibitors.

The preferred strategy for the development of MA-containing drugs involves the binding of non-catalytic cysteine residues with acrylamides and other α, β-unsaturated carbonyl compounds. There is a growing consensus that the covalent binding of target proteins using MA residues can improve pharmacodynamic properties such as the efficacy, potency, selectivity and duration of pharmacological effects. However, to reduce toxicity, the development of such inhibitors requires careful, thorough and sophisticated drug design, often using computational and molecular modeling methods.

Designed covalent inhibitors can offer significant advantages over non-covalent inhibitors, as covalent warheads can target the single residues of selected target proteins with higher pharmacodynamic efficacy and lower susceptibility to drug resistance. Therefore, in addition to traditional biomedical drugs, covalently binding inhibitors based on MA residues could become important players in the 21st-century drug market.

The anti-cancer properties of MA are closely linked to their chemical structure and reactivity. They exert their biological activity through electrophilic groups in their structure that can target nucleophilic residues on disease-relevant proteins, leading to significant therapeutic effects with low toxicity in many diseases. These compounds can be structurally modified to react selectively with nucleophilic targets or to be converted into selective MA during metabolism. Most of them exert their effects through pathways such as the Keap1-NRF2-ARE pathway and the NF-κB pathway and exhibit antioxidant and anti-inflammatory activities. Further research is needed to fully understand their potential and develop effective therapies.
